# A Systematic Review of Integrated Functional Near-Infrared Spectroscopy (fNIRS) and Transcranial Magnetic Stimulation (TMS) Studies

**DOI:** 10.3389/fnins.2019.00084

**Published:** 2019-02-28

**Authors:** Adrian Curtin, Shanbao Tong, Junfeng Sun, Jijun Wang, Banu Onaral, Hasan Ayaz

**Affiliations:** ^1^Drexel University, School of Biomedical Engineering, Science and Health Systems, Philadelphia, PA, United States; ^2^School of Biomedical Engineering, Shanghai Jiao Tong University, Shanghai, China; ^3^Shanghai Mental Health Center, Shanghai Jiao Tong University School of Medicine, Shanghai, China; ^4^Department of Family and Community Health, University of Pennsylvania, Philadelphia, PA, United States; ^5^Center for Injury Research and Prevention, Children's Hospital of Philadelphia, Philadelphia, PA, United States

**Keywords:** non-invasive brain stimulation (NIBS), functional near-infrared spectroscopy (fNIRS), transcranial magnetic stimulation (TMS), neuromodulation, cognition, motor, functional neuroimaging, TMS+fNIRS

## Abstract

**Background:** The capacity for TMS to elicit neural activity and manipulate cortical excitability has created significant expectation regarding its use in both cognitive and clinical neuroscience. However, the absence of an ability to quantify stimulation effects, particularly outside of the motor cortex, has led clinicians and researchers to pair noninvasive brain stimulation with noninvasive neuroimaging techniques. fNIRS, as an optical and wearable neuroimaging technique, is an ideal candidate for integrated use with TMS. Together, TMS+fNIRS may offer a hybrid alternative to “blind” stimulation to assess NIBS in therapy and research.

**Objective:** In this systematic review, the current body of research into the transient and prolonged effects of TMS on fNIRS-based cortical hemodynamic measures while at rest and during tasks are discussed. Additionally, studies investigating the relation of fNIRS to measures of cortical excitability as produced by TMS-evoked Motor-Evoked-Potential (MEP) are evaluated. The aim of this review is to outline the integrated use of TMS+fNIRS and consolidate findings related to use of fNIRS to monitor changes attributed to TMS and the relationship of fNIRS to cortical excitability itself.

**Methods:** Key terms were searched in PubMed and Web-of-Science to identify studies investigating the use of both fNIRS and TMS. Works from Google-Scholar and referenced works in identified papers were also assessed for relevance. All published experimental studies using both fNIRS and TMS techniques in the study methodology were included.

**Results:** A combined literature search of neuroimaging and neurostimulation studies identified 53 papers detailing the joint use of fNIRS and TMS. 22/53 investigated the immediate effects of TMS at rest in the DLPFC and M1 as measured by fNIRS. 21/22 studies reported a significant effect in [HbO] for 40/54 stimulation conditions with 14 resulting an increase and 26 in a decrease. While 15/22 studies also reported [HbR], only 5/37 conditions were significant. Task effects of fNIRS+TMS were detailed in 16 studies, including 10 with clinical populations. Most studies only reported significant changes in [HbO] related measures. Studies comparing fNIRS to changes in MEP-measured cortical excitability suggest that fNIRS measures may be spatially more diffuse but share similar traits.

**Conclusion:** This review summarizes the progress in the development of this emerging hybrid neuroimaging & neurostimulation methodology and its applications. Despite encouraging progress and novel applications, a lack of replicated works, along with highly disparate methodological approaches, highlight the need for further controlled studies. Interpretation of current research directions, technical challenges of TMS+fNIRS, and recommendations regarding future works are discussed.

## Introduction

Since its introduction in 1985 by Barker (Barker et al., 1985), Transcranial Magnetic Stimulation (TMS) has grown to be an effective tool in both research and in the clinic. With the use of an electromagnetic coil, TMS produces a brief but powerful magnetic field (1.5–2 Tesla) capable of inducing current and triggering action potentials within neurons of the superficial areas of the cerebral cortex (Valero-Cabré et al., 2017). In addition to transiently inducing or disrupting neural activity, the application of repeated TMS (rTMS) is capable of either facilitating cortical excitation or cortical inhibition via the mechanisms of Long-Term Potentiation (LTP) and Long-Term Depression (LTD), respectively (Pell et al., 2011). Certain rTMS paradigms have been shown to continue to influence neural behaviors over an hour after the stimulation period (Huang et al., 2005), while the behavioral effects of repeated stimulation have demonstrated the potential to last several weeks (Lefaucheur et al., 2014).

The prospect of manipulating not just instantaneous neural activities, but long-term behavior of neural populations is attractive to researchers who want to promote the recovery of damaged or disordered neural systems and enhance the function of existing networks. TMS therapy has already been approved by the FDA for use in unipolar depression (Major Depressive Disorder, MDD) (George et al., 2013) and a considerable amount of research effort is currently being devoted to identifying its utility in stroke rehabilitation (Langhorne et al., 2011), schizophrenia (Cole et al., 2015), phobia (Notzon et al., 2015), epilepsy (Kimiskidis, 2016), and many other conditions. TMS offers substantial promise as a cognitive probe and as a therapeutic technique. However, individuals may vary substantially in their responses to stimulation, and understanding of the explicit effects of TMS remains limited.

Current knowledge regarding the effects of TMS paradigms is based primarily on the physiological effect on an individual's resting motor threshold (RMT), i.e., the required TMS stimulation level in the Motor Cortex (M1) to elicit a motor evoked potential (MEP) 50% of the time. Identification of an individual's RMT is an important first step in calibrating TMS stimulation and changes in RMT which occur following repeated stimulation are thought to reflect changes in cortical excitability (Valero-Cabré et al., 2017). Trains of low frequency (1 Hz) stimulation have been shown to decrease cortical excitability (increase in RMT) and trains of high frequency stimulation (>5 Hz) have been shown to increase cortical excitability (decrease in RMT) (Fitzgerald et al., 2006). Theta burst stimulation (TBS) represents an additional paradigm in which short 3 pulse bursts of stimulation at 50 Hz are repeated at 5 Hz which is thought to have a more pronounced effect compared with High Frequency and Low Frequency stimulation (Huang et al., 2005). When stimulated in short intervals, intermittent TBS (iTBS) is thought to have a faciliatory action on cortical excitability and when stimulated continuously (cTBS), the paradigm is thought to be inhibitory. As a majority of accessible TMS measurements are produced from stimulation of the motor cortex, the principles learned from stimulation of this area are assumed to apply in other cortical regions.

Although studies certainly benefit from the knowledge of motor-cortex sensitivity to TMS stimulation, many regions of therapeutic and psychiatric interest are located in cortical regions which have no easily measured physiological response. In these regions, the efficacy of this “blind” neurostimulation can only be measured in terms of the behavioral changes induced, which may only be apparent after repeated stimulation sessions. It is here that neuroimaging techniques provide a practical solution to “close the loop” and evaluate the immediate (online) and integrated (offline) responses to TMS stimulation, potentially enabling the identification of optimal treatment paradigms through the measurement of individual responses and additional insight into the ways in TMS meaningfully affects behavior and cognition.

The combined use of TMS and neuroimaging has become an exciting new landscape on which to test theories of both low-level and complex cognitive functions as well as inform TMS-based therapies. The use of Electroencephalography (EEG), functional Magnetic Resonance Imaging (fMRI) and Positron Emission Tomography (PET) in multimodal TMS has been the subject of a number of comprehensive reviews (Bestmann et al., 2008; Reithler et al., 2011; Bortoletto et al., 2015; Hallett et al., 2017). Researchers have also employed functional Near Infrared Spectroscopy (fNIRS), an optical brain imaging technique which offers a number of benefits over other approaches. While each of these modalities offers particular advantages and disadvantages in terms of cost, spatial, and temporal resolution, fNIRS-based measurements are not intrinsically subject to electromagnetic interference and represent an affordably scalable technique to study both the immediate and prolonged effects of TMS. Although works have detailed the challenges and methodological issues associated with the use of concurrent TMS-fNIRS (Parks, 2013) and a number of studies have been published employing this approach, no systematic synthesis of this research is currently available. The purpose of this review is to consolidate studies related to the use of fNIRS and TMS in order to provide an accessible summary of the paradigms used, research questions addressed, and the current consensus on findings.

### TMS-fNIRS Integration and Challenges

fNIRS is a non-invasive brain imaging technique that takes advantage of the “optical window,” the natural transparency of tissue to near-infrared light (650–900 nm), to provide measurement of the changes in cortical hemoglobin concentrations (Villringer et al., 1993). Deoxygenated hemoglobin [HbR] and Oxygenated hemoglobin [HbO] are among the largest varying absorbers of light in the near-infrared spectrum and therefore the changes in light intensity can be used to estimate changes in chromophore concentration via the modified Beer-Lambert Law. fNIRS measures neural activities through the hemodynamic changes which occur due to neurovascular coupling (Fuster et al., 2005) and increases in neuronal firing rates in cortical areas are typically observed together with proportional changes in hemodynamic response (Heeger and Ress, 2002). Although the immediate effect of TMS is electromagnetic stimulation, the relatively tight coupling of neural activation to hemodynamic changes allows fNIRS to measure the effects of TMS (Allen et al., 2007). Despite this, the effects of TMS on local neuronal populations may depend significantly on the parameters employed, the location of the stimulation and the type and angle of the coil (Pashut et al., 2011). Additionally, the mechanisms of how individual TMS parameters impact hemodynamic response via excitatory and inhibitory synaptic activity remain to be determined (Arthurs and Boniface, 2002).

Since fNIRS measurements are based on the optical properties of the investigated medium, they are not fundamentally subject to electromagnetic interference produced by TMS coil operation and additionally do not place any restrictions on the placement of the coil. Alongside compatibility with TMS, fNIRS systems may be easily integrated with electrical stimulation approaches such as transcranial direct current stimulation (tDCS) (McKendrick et al., 2015), and can employ probes which are portable, wireless, well-tolerated, cost-effective, and can be applied in many situations in which subject movement is generally unrestricted including outdoor environments (McKendrick et al., 2016) and even vehicle operation (Gateau et al., 2018).

Despite these advantages, fNIRS has several considerations that must be addressed when used with TMS. fNIRS-based systems measure at depths that are a function of the optode distance between the light source and detector used (typically 2.5–4 cm), which effectively restricts the use of fNIRS measurements to shallow cortical regions (Okada and Delpy, 2003). Although sufficient for use with TMS, the spatial resolution of fNIRS is not as high as fMRI and the temporal speed of the measured hemodynamic response is much slower to evolve than the electrophysiological response measured by EEG. Integration with TMS may also impose restrictions on how and which fNIRS sensors are used. Although optically-measured fNIRS signals are not susceptible to electromagnetic interference, care must be taken to ensure that individual fNIRS systems are not only noise-free under TMS operation, but critically that these systems are properly shielded so as to prevent hardware damage from electromagnetic interference. Since TMS electrical fields degrade very quickly with distance, these issues are most important with LED-based or wireless systems that feature electronics localized closely with the coil.

TMS stimulation is typically accompanied by a vibration of the coil and an iconic clicking sound. While the motion associated with the TMS vibration is very limited and brief, fNIRS optode arrangements that include a TMS coil with close proximity may introduce mechanical noise into the signal or displace a component entirely. Typical fNIRS arrangements are also sensitive to both voluntary and involuntary movement of the subject during experiments which may result as a natural response of the individual to stimulation. Both of these situations can induce potentially large changes in the measured signal which are non-cortical in nature. Finally, fNIRS-based measurements may be subject to changes in the scalp caused by either TMS-induced stimulation of musculature or direct effects of TMS on superficial microvasculature. A variety of fNIRS techniques such as short-separation detectors have been proposed to provide measures of superficial blood flow in an attempt to resolve these issues (Gagnon et al., 2011). Typically, experimenters attempting to take advantage of combined TMS-fNIRS have addressed these issues by either not colocalizing the coil with sensors (measuring at a location distant from the coil), placing the coil above the fNIRS montage and increasing the power substantially to account for the weakening of the magnetic field with distance, designing a custom coil or optode arrangement that can be closely integrated together without interference, or simply choosing to measure offline. For a well-written description of technical issues concerning fNIRS and TMS integration the reader is referred to Parks (2013).

While there are multiple technical challenges associated with the integration of fNIRS and TMS, the combined approach of the two techniques offers a practical and flexible approach to studying the dynamics of cortical neurostimulation. In addition to ongoing development of newer fNIRS sensor technology and methods to improve signal quality, there exists a need for well-controlled studies which can verify and characterize the response of TMS-fNIRS on cortical activity at rest or during task. This review attempts to organize the current body of published work to provide an overview of current approaches, findings, and the degree of agreement between them. Although considerable heterogeneity in application and approach make direct comparison between individual studies difficult, we hope that the work here will encourage and contribute to future research directions. To this end, a systematic search has been conducted using Web Of Science and PubMed to collect, categorize, and consolidate multimodal TMS-fNIRS studies according to the primary stimulation sites investigated (M1 and DLPFC) and provide a narrative overview of this multimodal approach in the context of task and excitability applications.

## Materials and Methods

In order to provide a comprehensive and structured review of studies using fNIRS and TMS in combination, this review was conducted according to PRISMA recommendations (Liberati et al., 2009).

### Eligibility Criteria

Articles which reported the use of fNIRS and TMS as experimental techniques in their protocol and results were included, provided the article was published in a peer-reviewed journal and available in English. Both controlled and exploratory studies were included as eligible for this work and no restrictions were placed on the publication date of the studies. Studies which investigated any population group were at any age or gender were considered in this work. Works were excluded if they were non-experimental in nature (review, commentary, or purely methodological works), did not pertain to neuroscience as a discipline (out of field), did not involve cortical TMS stimulation or fNIRS, or were not in English. Conference papers were not considered eligible.

Primary outcomes of interest in this work were the type (HbO/HbR/HbT) and direction (increase/decrease) of fNIRS-biomarkers in the ipsilateral and contralateral hemisphere in response to TMS/rTMS delivered at rest, or prior to tasks. Cortical excitability papers were collected and summarized but not assessed in this manner.

### Information Sources

Research articles were located using the Pubmed MEDLINE and Web of Science, but additional works were referenced through the use of Google Scholar and a thorough study of the referenced works in other identified papers.

### Literature Search and Data Extraction

**Figure 1:**
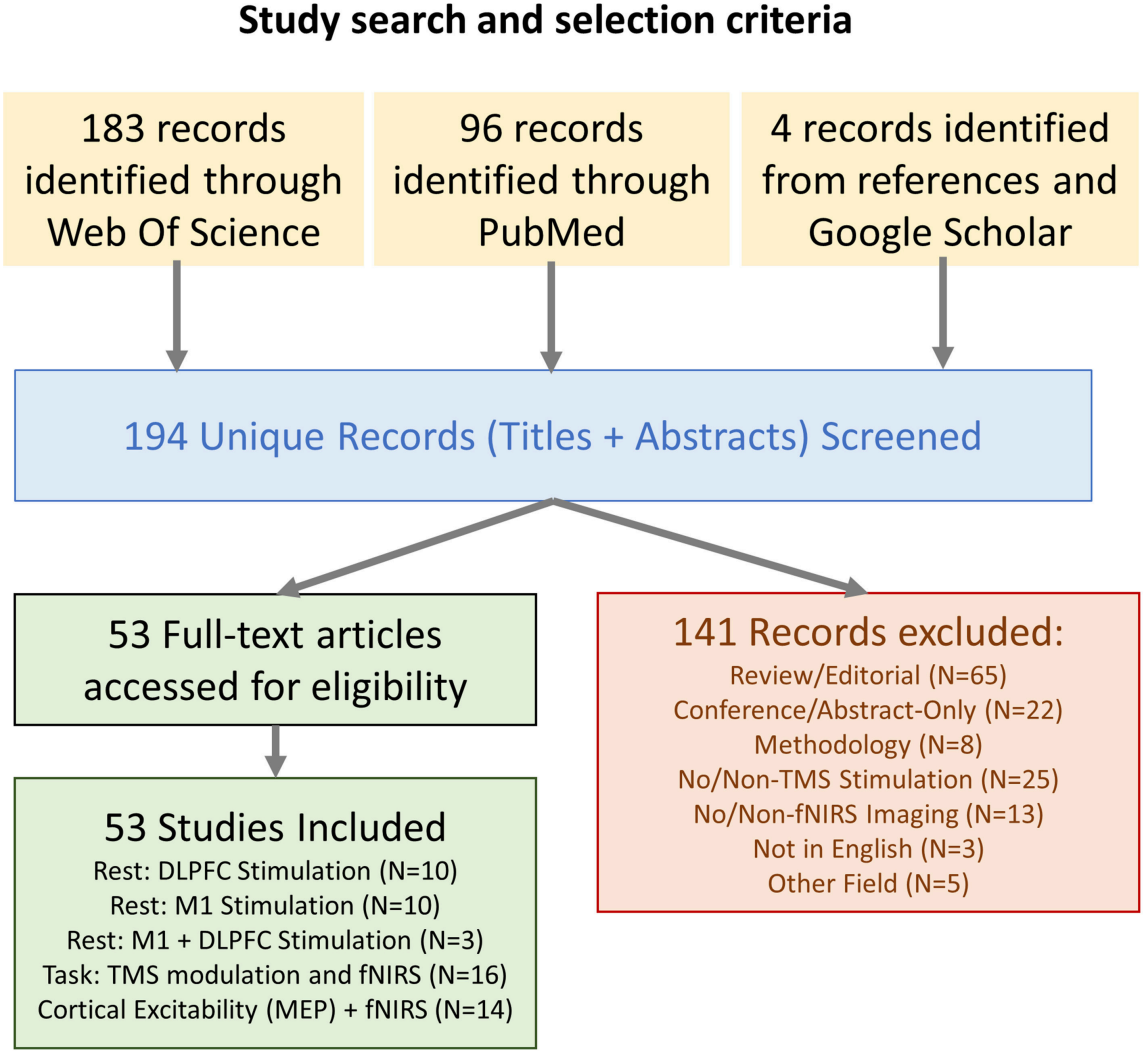
Flow chart describing the study selection.

All combinations of the following Medical Subject Headings (MeSH) were used in a Pubmed literature search, and the search was repeated as Subject Topics in a parallel Web Of Science search: (“fNIRS” OR “NIRS” OR “Near Infrared Spectroscopy” OR “Optical Topography” OR “Diffuse Optical Imaging”) AND (“TMS” OR “rTMS” OR “Transcranial Magnetic Stimulation”). References within articles identified as relevant and additional studies gathered from Google Scholar using similar search queries were also included. Studies were limited to non-conference academic publications published in English. The PRISMA chart for the screening and selection process is shown in Figure 1. The results and limitations of each study were assessed. The last date for this literature search was November 28th, 2018.

Two researchers (AC and HA) screened potentially relevant records on the basis of their titles and abstracts. The full text of each candidate article was accessed and reviewed to determine its eligibility. Information was extracted from the work regarding the subject population, number of subjects recruited, employed task, stimulation parameters, sham type, and location, as well as measurement area and primary outcomes. Primary results and conclusion of articles were identified, and discrepancies were resolved through discussion. Studies were arranged according to publication date and are here divided into ‘stimulation at rest’ with subdivisions based on the area of stimulation, ‘effects of rTMS on task’ with subdivisions based on healthy or clinical populations, and investigations into ‘fNIRS and cortical excitability’ with subdivisions for functional mapping, task-related excitability changes, and the study of central fatigue during exercise.

All information collected in the process of this review was organized within spreadsheets which contain the extracted information pertaining to each group. Attributes of each study were extracted; however, extracted information differed between the categories used to organize their review. For works which involved stimulation at rest the following information was extracted: stimulation parameters used, stimulation areas that were targeted, areas measured with fNIRS, whether fNIRS was measured online or offline, the type of sham stimulation employed, number of subjects, and a summary of findings. Additionally, results per stimulation condition, where applicable, were assessed for qualitative direction of change for typical biomarkers [HbO], [HbR], and [HbT] in ipsilateral and contralateral M1 or DLPFC to allow for a quantitative summary of results (detailed in Supplementary Table 1). For studies investigating the use of fNIRS to monitor either the effects of rTMS on tasks (as part of a clinical or non-clinical application), similar information was extracted with the addition of applicable task information (detailed information provided in Supplementary Table 2). For studies using MEP as a functional measure, the following information was extracted: population measured in the study, task employed/monitored with fNIRS, stimulation parameters used, stimulation area that was targeted, areas measured with fNIRS, whether TMS-fNIRS was measured online, type of sham stimulation employed, number of subjects in each experimental group, and a summary of findings. As these studies did not examine the impact of TMS or rTMS on fNIRS measures, qualitative assessment of changes in fNIRS biomarkers was not performed.

Due to the high heterogeneity of works currently available, extraction of statistical results from individual studies was not performed. Individual works differed substantially in protocol design, signal processing, stimulation methods, fNIRS equipment, subject population, and statistical approaches.

Several studies in this work also feature the Active Motor Threshold (AMT), a measurement which is similar to RMT except that the muscle is tonically active at 10% maximum voluntary contraction during testing (Terao et al., 1998). For the purposes of comparison, AMT has been converted to an estimation of RMT using the conversion factor (100%RMT = 140%AMT) and noted using a tilde (~) where this estimation has been performed.

## Results

### Study Selection

A total of 194 unique articles were identified in the initial search query, and 4 additional works were introduced which did not appear in the initial search but were identified as relevant through supplementary searches in Google Scholar and examination of references in the identified studies. Of these works, 141 were excluded after reviewing the title and abstract. This list included 65 articles that were either review or editorials, 22 conference articles/abstracts, 8 articles describing methodologies with no experiment, 25 articles which did not use TMS as part of the methodology, 13 articles which did not feature fNIRS-based neuroimaging, 3 articles which were not available in English, and 5 articles which related to other disciplines.

**Figure 2:**
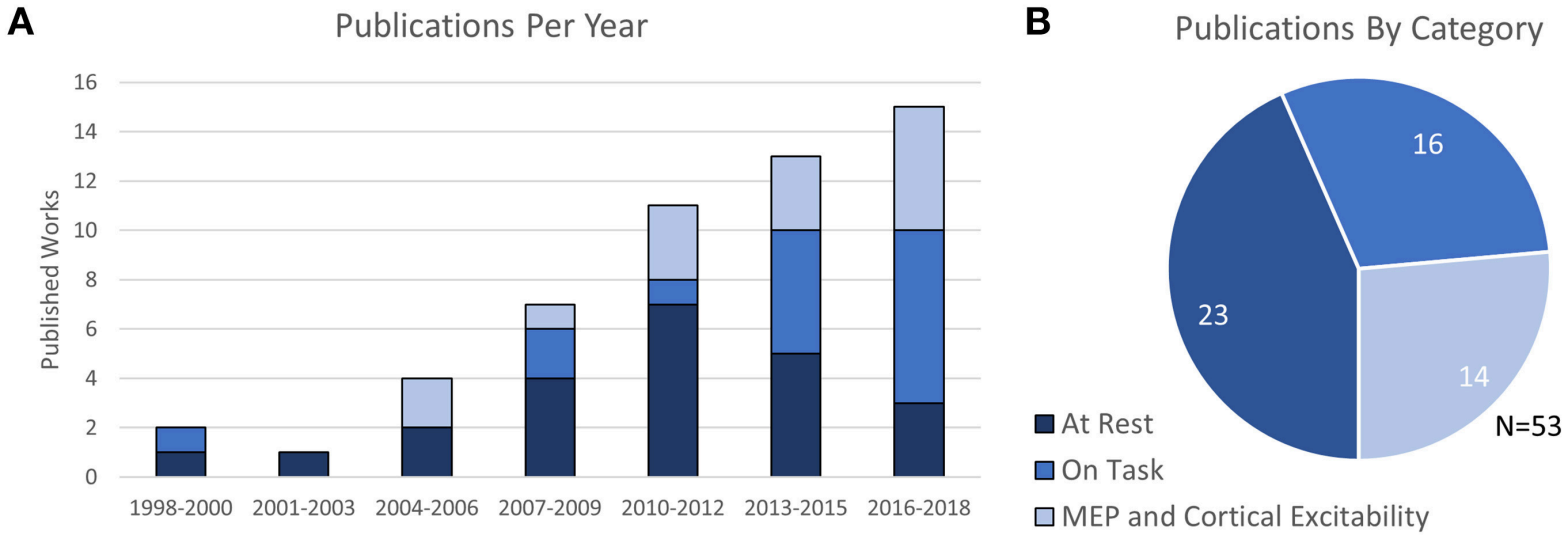
Number and Type of TMS-fNIRS studies **(A)** over time and **(B)** as categorized.

In total, the 53 selected works were broadly categorized into four groups depending on their content: 10 works were categorized as Prefrontal Stimulation at rest, 10 works were categorized as Motor Cortex Stimulation at rest, 3 works were categorized as both Prefrontal and Motor stimulation at rest, and the remaining 30 publications were categorized as either fNIRS-measured effects of TMS on cognitive tasks or studies which related fNIRS-measures to TMS-measured cortical excitability (MEP). The trend of publication over time and distribution of categorized work is shown in Figure 2.

### Review Organization

In the first section of this review, collected works were organized to investigate the effects of TMS on cortical activity as measured by fNIRS to compare the responses at different cortical regions at rest. Since the majority of publications involving TMS-fNIRS have focused on stimulation to the DLPFC and M1, these regions have been specifically discussed in separate sub-sections below.

In the second section of this review, the effects of TMS on task-evoked fNIRS measures is investigated and discussed within the study-specific contexts. These works feature studies in which rTMS may be applied just prior to a specific task or as stimuli within a task with the expectation that TMS will influence either task performance or cortical involvement in a manner measurable by fNIRS. As rTMS in these situations was often applied as part of a therapeutic strategy for clinical disorders, studies featuring healthy and clinical populations are described in separate sections.

In the final section, works focusing on the relationship of the cortical excitability measures and fNIRS are discussed. The first sub-section focuses on the works using a combination of TMS measures and fNIRS as complementary techniques in functional motor mapping. The second sub-section introduces works discussing how TMS-measured cortical excitability may change during task performance and its relationship to similarly measured task-evoked fNIRS. Finally, works investigating the use of fNIRS and TMS-measured cortical excitability under the context of exercise physiology are presented. These studies primarily focus on the use of fNIRS as a measure of physiological influence on central fatigue due to hypoxia and other conditions.

## Effects of TMS Stimulation on fNIRS Measures at Rest

### Motor Cortex (M1)

**Figure 3:**
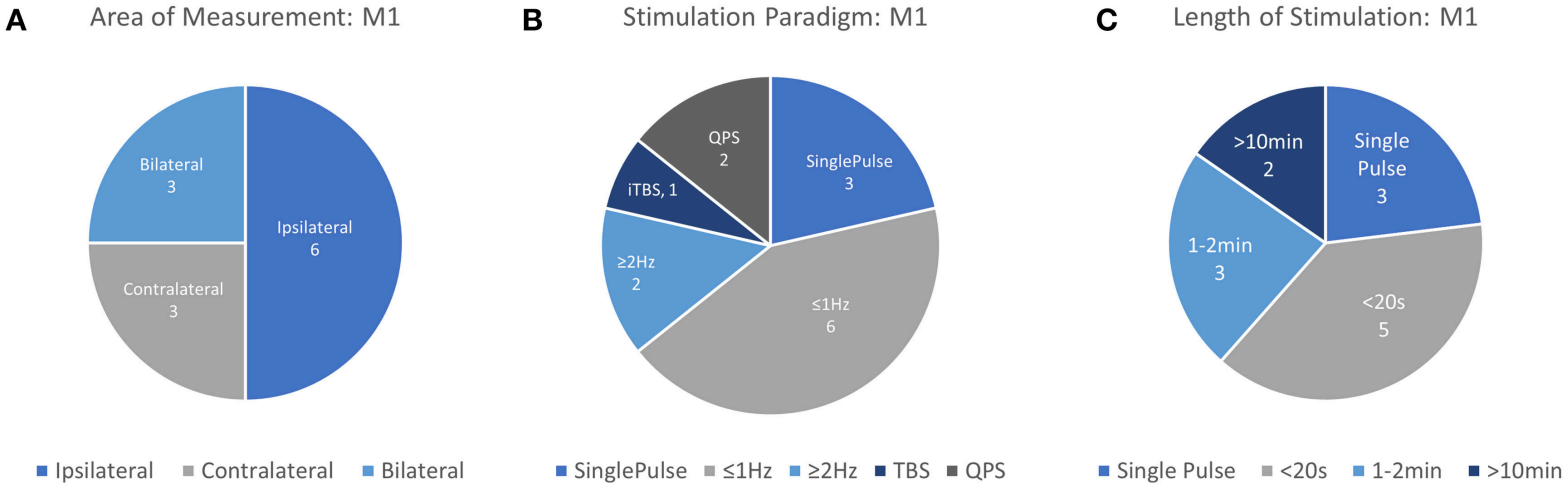
Details of TMS-fNIRS studies applied at rest to M1 for **(A)** Hemisphere measured, **(B)** Stimulation paradigm, and **(C)** Length of stimulation.

Due in part to the availability a quantifiable response in the form of MEP measurement, motor cortex behavior provides an accessible means to understand the effects of transient and repeated stimulation by TMS. However, the application of TMS in this cortical area also has important clinical applications. Single pulses of TMS are approved by the FDA in the US as a prospective tool for neurosurgical planning in the primary motor cortex. In this procedure, physicians noninvasively disrupt active cortical circuits in patients preparing to undergo neurosurgery as an alternative to direct cortical stimulation which requires a craniotomy (Eldaief et al., 2013). These stimulations can help surgeons identify areas which may be preferentially avoided to prevent complications and disability resulting from neurosurgery. Additionally, rTMS methods have been proposed as a therapeutic technique for a number of motor-related disabilities including Parkinson's disease and stroke. Even with the accessibility of MEPs, passive neuroimaging offers a more continuous method of measuring cortical properties across multiple brain areas with the additional promise of sensitivity to stimulation which occurs below the motor threshold. Therefore, researchers continue to study the motor cortex in order to understand the influence of TMS on other neurophysiological metrics such as those measured by fNIRS. Details of each study targeting M1 including stimulation parameters, measurement area, primary findings, and other information are summarized in Table 1 and visualized in Figure 3.

**Table 1:** Studies investigating fNIRS-measured response to TMS stimulation of M1.

**References**	**Stimulation parameters**	**Stimulation area**	**Measurement area**	**Sham**	**No. of subjects**	**Finding**
Oliviero et al., 1999^*^	0.25 Hz, 100% Stimulator Power, 2 min	R-M1	R-M1 (Offline)	None	4	[HbO] increase vs. baseline after 30 stimulations
Noguchi et al., 2003	Single Pulse, {50%, 64%, 79%RMT} × 20 Trials	L-M1	L-M1	None	6	[HbO] increase for 79 and 64% RMT, no change for 50%RMT
Mochizuki et al., 2006	Single Pulse, {50%, 64%, 79%RMT} × 20 Trials {Active contraction, Relaxed}	L-M1	L-M1	Distant coil + electrical stimulus	8	[HbO] increase at 50% RMT when FDI contracted, decrease in [HbR] when 79%RMT [HbR] decrease at 64 and 79% RMT when at rest
Hada et al., 2006	{(0.5 Hz,20 s), (2 Hz,5 s)}, {80%, 120%RMT}, × 10 Trials	L-M1	L-M1	None	12	[HbO] decrease for 1 Hz and 2 Hz, larger decrease for 120% than 80%RMT
Mochizuki et al., 2007	2 s, iTBS (30 pulses), {57%, 71%RMT}	L-M1, L-S1, L-PM	R-PFC R-PM, R-M1, R-S1	Distant Coil	8	At 57%RMT: [HbO] decrease in contralateral PM when stimulated in PM, [HbO] decrease in contralateral S1 when M1 stimulated [HbO] decrease in contralateral M1 and S1 when S1 stimulated
Kozel et al., 2009^*^	1 Hz, 120%RMT, 10 s × 15 trials, 2 Days	L-M1	Bilateral M1	None	11	[HbO] decrease in ipsilateral and contralateral M1
Tian et al., 2012^*^	1 Hz, 120%RMT, 10 s × 15 trials, 2 Days	L-M1	Bilateral M1	None	11	Reliability Assessment of (Kozel et al., 2009)
Näsi et al., 2011	{0.5, 1, 2 Hz}, 75%RMT, 8 s × 25 trials	L-M1, Shoulder	Bilateral M1, Bilateral Shoulders	None	13	[HbT] decrease in bilateral M1, strongest at 2 Hz [HbT] decrease on stimulated shoulder, increase on opposite shoulder, correlations with PPG, HR
Hirose et al., 2011	{QPS-5,QPS50} at 0.2Hz, 79%RMT, 2 min X3 trials	L-M1	R-PM, R-M1, R-S1	Distant Coil	9	Decrease in contralateral [HbO] during stimulation for QPS-5 in measured areas and QPS-50 in M1
Groiss et al., 2013	Exp1: {QPS-5,QPS50} at 0.2Hz, 64%RMT, 2 min X3 trials Exp2: {QPS-5, QPS-50}, 64%RMT × 10 Trials	L-M1	L-M1,L-S1,L-PM, L-SMA,L-PFC	Distant Coil	Exp1:10 Exp2:7	[HbO] decrease in ipsilateral M1 for rQPS-5, single QPS-5 burst reduced [HbO] in M1 and PM, no change for QPS-50
Furubayashi et al., 2013	Single Pulse, {50%, 64%, 79%RMT} × 20 Trials {Active contraction, Relaxed}	L-M1	L-M1	Distant Coil	15	[HbO] increase during stimulation, increases with stimulation power in both active and relaxed condition [HbO] decrease 10s after stimulation in active condition, magnitude increases with stimulation power
Mesquita et al., 2013	1 Hz, 95%RMT, 20 min	L-M1	Bilateral M1	None	7	[HbO] increase ipsilaterally during stimulation, increase [CMRO2], no change contralaterally
Park et al., 2017	1 Hz, 90%RMT, 20 min	L-M1	R-M1, R-PM	Distant coil	11	[HbO] increase contralaterally in M1, PM1 and decrease in [HbR], smaller response in PM than M1

* *Indicates that the study appears in both M1 and DLPFC tables*.

The first use of fNIRS and TMS together was reported by Oliviero et al. (1999). The authors examined the impact of 0.25 Hz stimulation repeated for 2 min (30 pulses) at maximum stimulator power (100% machine power) over the right motor cortex (M1) as well as the right DLPFC. While this stimulation strength is uncommonly high and unrelated to participant RMT, the authors reported that the effective stimulation strength was reduced by the added distance of the interceding fNIRS probe The authors observed a post-stimulation increase in [HbO] after 2 min of stimulation in both regions with a slightly larger magnitude of response in R-M1 than the R-DLPFC. However, the results of this first study may be incomparable to other works due to offline measurement, unknown effective stimulation strength, uncommon stimulation frequency, and in particular, a rather small subject size for the measure of M1 (*N* = 4). The first online work using fNIRS was reported in a conference paper by Nissilä et al. (2002), which examined the contralateral response to 30 s of 110%RMT stimulation at 0.5 Hz (15 pulses) over left-M1. Despite a limited number of subjects and the use of only a single NIR wavelength, the authors concluded that a decrease in absorption at 830 nm reflected an increase in [HbO] vs. sham and that the area of greatest increase during finger tapping matched the region of contralateral activation.

The first colocalized work with fNIRS was conducted by Noguchi et al. (2003), examining the effect of single pulses of TMS over left-M1 at various stimulation levels (~50, ~64, and ~79%RMT) and observed that both 64 and 79%RMT resulted in an increase in [HbO]. This work was followed up by an additional sham-controlled experiment wherein subjects were in a relaxed state or tonically contracting the first dorsal interosseous muscle (FDI) (Mochizuki et al., 2006). Authors observed that stimulating at 50%RMT during contraction increased [HbO], but when relaxed, both 64 and 79% RMT resulted in large decreases in [HbR]. Using an integrated TMS-fNIRS coil, Furubayashi et al. (2013) replicated the work of Mochizuki et al. (2006) presenting stronger evidence for an increase in [HbO] associated with single pulse stimulation during relaxed and active conditions, representing an important verification of claims regarding single pulse stimulation in the motor cortex.

Hada et al. (2006) studied the response of fNIRS to short trains (10 pulses) of 0.5 and 2 Hz stimulation at 80% and 120%RMT in left-M1. The authors suggested that all conditions resulted in a decrease in [HbO] partnered with a slight increase in [HbR] and that changes increased with stimulation strength. Mochizuki et al. (2007) measured the effects of 2 s of excitatory theta burst stimulation (iTBS, 30 pulses) at ~57 and ~71% RMT over the left premotor (PM), M1, and primary sensory (S1) areas. The authors observed that iTBS stimulation at 57%RMT typically decreased [HbO] in the contralateral region of stimulation, but that the response varied by region stimulated. Here, M1 showed a decrease in [HbO] when contralateral S1 was stimulated, but in S1, [HbO] decreased when either the contralateral M1 or S1 was stimulated. The authors attributed this to an increased interhemispheric directional connectivity between M1 and S1, relative to PFC, which did not change in response to any regional stimulation, and PM, which only responded when contralateral PM was targeted.

Further work by Kozel et al. (2009) examined the bilateral responses to stimulation at either the left-M1 or left-DLPFC using 10 pulse trains of 1 Hz stimulation at 120%RMT. The authors found that short 1 Hz trains resulted in a bilateral [HbO] decrease in M1 and found a similar bilateral response in the DLPFC when stimulating the left-DLPFC. In a later report, the authors examined the characteristics of these responses and found that they had high spatial reproducibility (Tian et al., 2012).

Näsi et al. (2011) examined the contribution of physiological parameters to the fNIRS measured response of TMS by short trains of stimulation. The authors observed a bilateral decrease in [HbO] and [HbT] after 8 s of 75%RMT stimulation at 2 Hz over M1, but cast doubt that this change resulted from purely cerebral sources by pointing out strong correlations with photoplethysmogram (PPG) and heart rate (HR). In order to compare the changes attributed to cortical stimulation with systemic changes due to non-cortical stimulation, they conducted an experiment in which the fNIRS-measured TMS response was recorded again with stimulation and measurement occurring on the shoulders instead. In this set of experiments, they again observed that stimulation of the shoulder resulted in a decrease in [HbT] and an increase in the contralateral shoulder. Although measured in this case using fNIRS, the effect of TMS on systemic and autonomic processes has been observed in several forms, including changes to the vasomotor reactivity (Vernieri et al., 2014). These components must be taken into account in any interpretation of TMS and its effects on hemodynamic neurocorrelates. Although TMS has not been known to have any immediate impact on vasoconstriction itself, this does not bar changes attributable to autonomic intermediaries or systemic responses to the act of stimulation itself.

An investigation into the effects of Quadripulse Stimulation (QPS) and long trains of QPS bursts was examined by Hirose et al. (2011). QPS stimulation is suggested to induce powerful potentiation when four pulses of TMS are delivered with an interstimulus interval (ISI) of 5 ms, whereas a powerful neural depression is induced with an ISI of 50 ms (Hamada and Ugawa, 2010). The contralateral cortical response of these two paradigms (QPS-5 and QPS-50) was examined during 2 min of 78%RMT stimulation. While the the QPS-5 condition produced a measurable decrease in [HbO] in all contralaterally-measured areas (M1,PM,S1), changes associated with QPS-50 stimulation were only observed in contralateral M1. A further study by the same group (Groiss et al., 2013) monitored the ipsilateral response to QPS in these regions at 64%RMT and observed that a 2-min train of QPS-5 reduced [HbO] in ipsilateral M1 whereas a single burst of QPS-5 reduced [HbO] in both M1 and the PM. Under the same protocol, QPS-50 at 64%RMT produced no observable changes under either the 2-min train or single burst condition.

By and large, the previously studied works examined responses to stimulation based on TMS paradigms not typically featured in rTMS-based therapies. Stimulation using clinically relevant protocols have only been studied in two contexts. First, Mesquita et al. (2013) recorded the changes in bilateral M1 in response to clinically relevant 20 min, 95%RMT, 1 Hz stimulation trains over left-M1. During the course of stimulation, an increase in ipsilateral [HbO] was observed which resolved back to baseline following the protocol completion. Additionally, the authors reported that the estimated ipsilateral cerebral metabolic rate of oxygen consumption (CMRO2) increased during stimulation. No changes were noted in the contralateral side of stimulation for either measure. In the second case, Park et al. (2017) performed a similar stimulation paradigm at a slightly lower power level (90%RMT) while measuring contralateral changes in M1 and P1. Here, 20 min of 1 Hz stimulation were observed to produce contralateral increases in [HbO] in both measured regions. Although ipsilateral regions were not measured, these two works highlight the potential variability in response to even largely similar stimulation protocols.

In total, 12 unique studies were assessed for qualitative evaluation of the fNIRS-measured response to TMS in M1 and consistent findings are summarized here. Of these works, 3 publications studied Single Pulse effects at different subthreshold stimulation power levels with 9 different conditions. 8/9 of these conditions resulted in either an increase in [HbO] (5/9) or a decrease in [HbR] (3/9) in ipsilateral M1, suggesting that in most conditions, single pulse stimulation produced a measurable increase in Hemoglobin Difference ([HbDiff] = [HbO]–[HbR]) with sufficient stimulation power. Excitatory rTMS bursts applied to M1 have been studied in only 3 works, and currently, there is no overlap between areas measured, paradigm used, or length of stimulation to allow any preliminary conclusions to be drawn. Inhibitory rTMS was studied by 6 works either as a short train or burst (4 studies), short session (2 studies), or long session (2 studies). Short trains (8–10 s) of both subthreshold and suprathreshold rTMS stimulation ranging from 0.5 to 2 Hz were noted to induce ipsilateral decreases in [HbO] in 8/8 conditions across 4 independent works with bilateral reductions noted whenever measured (4/4 conditions). Response of M1 to a 20-min session of subthreshold 1 Hz stimulation has been suggested to produce an increase in ipsilateral [HbO] or an increase in contralateral [HbO], but more must be done to substantiate these findings. More work needs to be done to replicate and standardize protocols attempting to measure even similar rTMS paradigms so more direct comparisons between works can be drawn. Notably, the response to single pulse stimulation in contralateral M1, responses to suprathreshold single pulse stimulation in M1, the effects of high frequency rTMS (>5 Hz) have not been examined using fNIRS.

### Dorsolateral Prefrontal Cortex (DLPFC)

**Figure 4:**
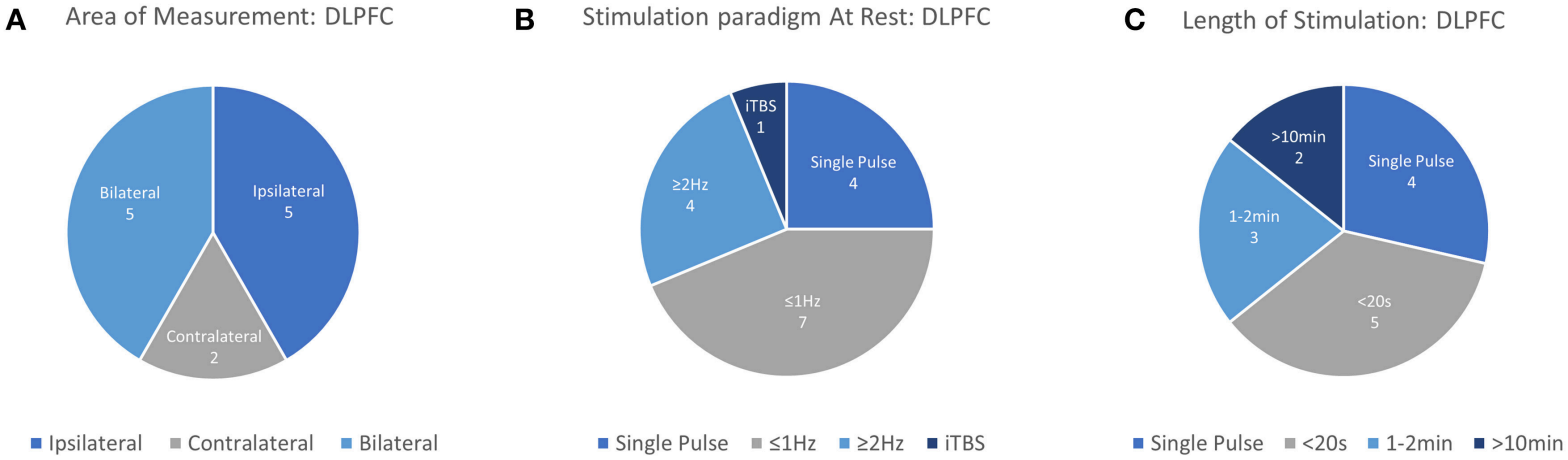
Details of TMS-fNIRS studies applied at rest to the DLPFC for **(A)** Hemisphere measured, **(B)** Stimulation paradigm, and **(C)** Length of stimulation.

The dorsolateral prefrontal cortex (DLPFC) is a critical area to many executive functions such as attention, working memory, response inhibition, problem solving. As an associative area and a key cortical area in many of the networks underlying complex cognitive function, the DLPFC is of great interest in the areas of cognitive science, neurology, and psychiatry. Apart from being a promising avenue of research, the use of rTMS targeting DLPFC for the treatment of MDD is currently the only FDA-approved use of rTMS in psychiatry. The therapeutic strategy for MDD prescribes the use of Low Frequency stimulation (~1 Hz) on the right-DLPFC (near F4) or High Frequency Stimulation (>5 Hz) over the left-DLPFC (near F3), with both paradigms demonstrating somewhat equivalent clinical efficacy (George et al., 2013). Despite being the only cortical region with an approved application for rTMS treatment, the lack of peripherally evoked responses to TMS stimulation in the DLPFC has spawned significant interest in finding neurophysiological measurements which can guide the application of TMS therapy. Here, neuroimaging techniques offer a quantifiable response to stimulation which allows a deeper understanding of TMS and rTMS-induced cognitive modulation. Information from each study investigating the DLPFC including stimulation area, measurement area, primary findings and other details are summarized in Table 2 and visualized in Figure 4.

**Table 2:** Studies investigating fNIRS-measured response to TMS stimulation of DLPFC.

**References**	**Stimulation parameters**	**Stimulation area**	**Measurement Area**	**Sham**	**No. of subjects**	**Finding**
Oliviero et al., 1999^*^	0.25 Hz, 100% Stimulator Power, 2 min	R-DLPFC	R-DLPFC (Offline)	None	10	[HbO] increase vs. baseline after 30 stimulations
Hanaoka et al., 2007	1 Hz, ~50%RMT, 60 s × 3 Trials	R-DLPFC	L-DLPFC	Distant Secondary Coil	11	[HbO] decrease during stimulation, subsequent increase
Aoyama et al., 2009	1 Hz, {28,41,58%}RMT, 60 s	R-DLPFC	L-DLPFC	Distant Secondary Coil	10	[HbO] decreases during rTMS when RMT > 50%
Kozel et al., 2009^*^	1 Hz, 120%RMT, 10 s × 15 trials, 2 Days	L-DLPFC	Bilateral DLPFC	None	11	[HbO] decrease in ipsilateral and contralateral PFC, [HbR] increased
Tian et al., 2012^*^	1 Hz, 120%RMT, 10 s × 15 trials, 2 Days	L-DLPFC	Bilateral DLPFC	None	11	Reliability Assessment of (Kozel et al., 2009)
Thomson et al., 2011a	Single Pulse, {90,110,130%} RMT × 15 trials	L-DLPFC	L-DLPFC	Distant Coil with electrical sham	12, 10 Sham	[HbO] decrease with 130%RMT, but not lower power
Thomson et al., 2011b	Single Pulse, 120%RMTPaired Pulse, (ICI,2 ms), (IFC 15 ms), 70%RMT conditioning, 120%RMT stimulus × 20 trials	L-DLPFC	L-DLPFC	Distant Coil with electrical sham	8, 10 Sham	[HbO] decrease with single pulse, SICF and SICI stimulation
Thomson et al., 2012a	{2 or 4 pulses}, 0.2 Hz, 130% RMT × 20 trials	L-DLPFC	L-DLPFC	Distant Coil with electrical sham	13, 10 Sham	[HbO] decrease for 2 pulses, larger decrease with 4 pulses
Thomson et al., 2012b	1 Hz, {80%, 120% RMT}, 10 min	L-DLPFC	L-DLPFC	None	6	Sustained [HbO] decrease for 10 min for 120%RMTSmall increase in [HbO] during 1st min for both 80 and 120%RMT
Thomson et al., 2013	Single Pulse, 120%RMT, {45,135,225 deg} × 15 trials 1 Hz, 120%RMT, 20 s, {45, 225 deg} × 15 trials	L-DLPFC	Bilateral DLPFC	None	12 SP, 8 rTMS	Ipsilateral [HbO] decrease for single pulse at 45 deg, Bilateral [HbO] increase for 20 s 1 Hz at 45 degrees, 135 deg orientation does not elicit significant response
Cao et al., 2013	{1, 2, 5}, 120%RMT, 5s × 20 trials	L-DLPFC	Bilateral DLPFC	None	12	[HbO] decrease for 1 Hz stimulation, increase for 2 Hz and 5 Hz
Curtin et al., 2017	{(1 pulse, 110%RMT), (15 Hz, 110%RMT, 2 s), (iTBS, 90%RMT, 2 s)} × 10 Trials	L-DLPFC	Bilateral DLPFC	Flipped Coil with stimulation	17	[HbO] increase for 15 Hz suprathreshold stimulation
Shinba et al., 2018	10 Hz, 120%RMT, 4 s × 75 trains, × 30 Days	L-DLPFC	Bilateral DLPFC	None	15 MDD	Increased [HbO] during stimulation associated with continued therapeutic effect of rTMS

* *Indicates that the study appears in both M1 and DLPFC tables*.

Oliviero et al. (1999) first examined the effects of rTMS in the prefrontal region alongside the previously discussed results in M1. fNIRS was used to measure changes following 2 min of rTMS stimulation delivered at 0.25 Hz and 100% Machine power to Fp2. These results were contrasted with the effects of anodal transcranial electrical stimulation (TES) and rTMS to right-M1. Again, the use of full TMS power is atypical and may represent as much as 300% RMT, but TMS strength would be reduced by distance created by the presence of the fNIRS probe between the cortex and the coil. Authors observed that levels of [HbO] had significantly increased following stimulation, similar to results observed in M1, while anodal electrical stimulation resulted in no significant change. Results from this study are difficult to compare due to the use of offline measurement, an uncommon stimulation frequency (0.25 hz), and the unknown stimulation strength resulting from an uncertain scalp-coil distance and lack of adaptation to subject RMT.

In the first online studies of stimulation in the DLPFC, Hanaoka et al. (2007) measured the effect of 1 Hz stimulation on the right-DLPFC (5 cm anterior to M1) while using fNIRS to monitor the contralateral DLPFC. In the first study, healthy subjects were instructed to idly copy a cartoon image while receiving stimulation at an effective 50%RMT to the right-DLPFC, whereas a second study by Aoyama et al. (2009) reported the effect of varying stimulation power with the same paradigm. Both studies suggested that 50% RMT was sufficient to produce a contralateral decrease in [HbO] during stimulation, while lower levels of stimulation were insufficient to effect significant change. Further work by Kozel et al. (2009) performed the first online ipsilateral measurements of stimulation to the DLPFC using 10 s of stimulation at 1 Hz with 120%RMT power. Although this work featured no sham condition, the work was counterbalanced with additional stimulation over the motor cortex. The authors reported that 1 Hz stimulation over the left-DLPFC (5 cm anterior to M1) was associated with large decreases of [HbO] in the bilateral DLPFC and in a later publication assessed the spatial reliability of these responses as having high reproducibility (Tian et al., 2012).

A fourth set of studies by Thomson et al. (2011a,b); Thomson et al. (2012a,b); Thomson et al. (2013) represents an attempt to characterize the response of the left-DLPFC (F3) to cortical stimulation. As the framework for several of these studies is largely similar, it is likely that they featured a common control subject set and a degree of overlapping participants. The first of six studies published investigated the ipsilateral response to single pulses of TMS at varying amplitudes (Thomson et al., 2011b) and found that only 130%RMT stimulation resulted in a large decrease in [HbO] and that neither 90%RMT or 110%RMT sufficiently produced a response. A second study (Thomson et al., 2011a) attempted to discern if fNIRS captured measurable differences between single pulses and paired pulses spaced at 2 or 15 ms, denoting stimuli for intracortical inhibition (ICI) and intracortical facilitation (ICF) respectively, two phenomena which are demonstrated to be sensitive to glutamate and GABA neurotransmission (de Jesus et al., 2014). They reported that single pulses at 120% RMT, as well as both ICI and ICF paired pulses, decreased [HbO] and suggested differences in the temporal dynamics of this decrease between the stimuli types. An additional study (Thomson et al., 2012a) investigated the differential response to two or four suprathreshold (120%RMT) TMS pulses spaced at 5 s, finding that both decreased [HbO], but four pulses had a stronger effect. Altogether these studies imply a general role for decreased [HbO] in response to strong suprathreshold stimulation and suggest that increased stimuli dosage may increase and prolong this response.

Thomson et al. (2012b) also featured a work recording the response of the left-DLPFC to long trains (10 mins) of 1 Hz stimulation typical of rTMS therapy. Again recording and stimulating over the left-DLPFC, the authors reported that 1 Hz at 120%RMT for 10 min produced a sustained decrease in [HbO]. However, the authors also noted that both 80%RMT and 120%RMT stimulations increased [HbO] during the first minute of stimulation. In addition to these works, Thomson et al. (2013) investigated the effect of coil orientation on the effect of single pulse stimulation and short trains of rTMS (20 s), noting primarily that the commonly used 45-degree orientation provided the strongest response and that a 135-degree coil orientation did not produce significant responses to stimulation. Similar to their previous findings, Single Pulses at 45 degree orientation and 120% RMT were again shown to have an ipsilateral decrease in [HbO]. However, 20 s of 1 Hz stimulation at 120%RMT was shown to instead increase [HbO] bilaterally. The authors noted that this increase in [HbO] may be an effect of cumulative stimulation as early rTMS trials showed decreased [HbO] and later trials showed larger increases.

The last work from this group, published by Cao et al. (2013), investigated the effect of 5 second trains of 1, 2, and 5 Hz stimulation at 120% RMT, showing again a decrease in [HbO] after 1 Hz and demonstrating an increase [HbO] after 2 and 5 Hz stimulation. In a recent publication, we similarly investigated the differential response to single pulses and short trains of rTMS (2 s) at 15 Hz conditions, as well as intermittent theta burst (iTBS) stimulation at 110%RMT and 90%RMT, respectively (Curtin et al., 2017). Our results suggested that single pulses at 110% RMT were not sufficient to elicit a response over the DLPFC, whereas 2-s trains of 15 Hz stimulation produced an increase in [HbO]. Subthreshold (90%RMT) trains of iTBS stimulation were not observed to introduce immediate changes in the area of stimulation despite claims of enhanced efficacy relative to high frequency stimulation.

Most recently, a clinical pilot study conducted by Shinba et al. (2018), reported significant increases in midline [HbO] during 10 Hz rTMS stimulation at 120%RMT to the left-DLPFC (5.5 cm anterior to M1) for treatment of drug-resistant individuals with MDD. Over the course of a 6-week treatment regime, the authors observed that the continued presence of [HbO] increase in response to stimulation was associated with clinical improvement as described by the Montgomery-Asberg Depression Rating Scale (MARDRS). Individuals who did not show positive changes associated with stimulation toward the end of the therapeutic regime did not show as strong clinical improvement.

In this review, 12 unique articles were identified which explored the effect of TMS on fNIRS-measured activation in the DLPFC. Of these articles, 4 works explored the effect of Single Pulse Stimulation in 6 conditions suggesting that single pulse stimulation of at least 120%RMT produce a measurable decrease in ipsilateral [HbO] (3/3 conditions). While the results of these works are in agreement with each other, the majority of these studies have been produced by one group and may not provide strong or independent evidence for this finding. The effects of inhibitory 1 Hz stimulation has been studied by 6 works, with 5/6 studies reporting a decrease in contralateral or ipsilateral [HbO] with sufficient stimulation power in at least one reported condition. Of these works, 2 early studies reported contralateral decreases in [HbO] with >50%RMT (while drawing), whereas suprathreshold 1 Hz stimulation at 110–120%RMT varied in reported effect. Here, short 5 and 10 s trains were independently reported to decrease ipsilateral or bilateral [HbO], while one study reported bilateral increase in [HbO] with 20 s stimulation. Only one study investigated longer clinically-relevant 1 Hz rTMS stimulation and reported [HbO] decrease only for suprathreshold (120%RMT) stimulation. Only 3 works investigated excitatory stimulation using rTMS, with two studies examining short (2–5 s) trains. These two independent studies suggest that short trains of suprathreshold high frequency stimulation produced either ipsilateral or a bilateral increase in [HbO]. While the remaining study measuring the clinical response of suprathreshold rTMS therapy in MDD provides some evidence for an expected increase in [HbO] and even a potential clinical correlate of treatment response, similar rTMS effects have not been described for healthy controls. In summary, despite evidence for decreased ipsilateral [HbO] in response to suprathreshold Single Pulse stimulation (3/4 studies), decreased ipsilateral or contralateral [HbO] in response to 1 Hz stimulation (5/6 studies), and increase [HbO] in response to suprathreshold excitatory high frequency stimulation (3/3 studies), a notable lack of consistency exists for specific parameters, protocols, precise stimulation target (F3 vs. 5 cm rule), and measurement areas used. This in combination with smaller subject sizes and a lack of well-controlled studies may limit interpretation of these results.

## Effects of rTMS Stimulation on Task-Evoked fNIRS Activity

**Figure 5:**
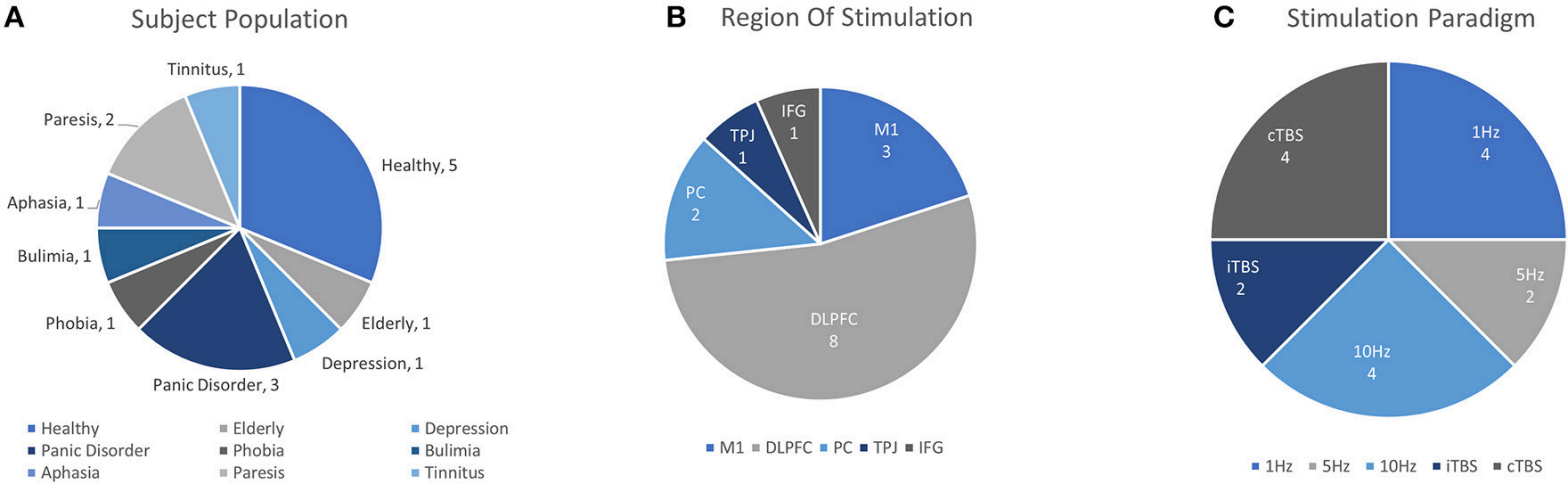
Details of TMS-fNIRS studies applied before or during cognitive Tasks according to **(A)** Subject Population, **(B)** Region of Stimulation, and **(C)** Paradigm of Stimulation.

The ability of rTMS to modulate cortical excitability promises an accessible, non-invasive way to facilitate cognitive function and treat disorders of brain function. To this end, researchers have become interested in the way rTMS paradigms effect changes on task performance and the associated neural activities involved, but also conversely, the way in which those activities may predict or reflect efficacy of rTMS itself. As changes in cortical excitability are largely inferred by effects on MEP (recorded while at rest in the motor region), it is often difficult to anticipate the way that rTMS paradigms will alter task-evoked measures of neural activity. Changes in functional measures during task-related activity are particularly important given interest in using rTMS to influence high-level cognitive function. Despite the potential utility of fNIRS for this purpose, this topic has been the subject of relatively few works. Although somewhat disparate in application and implementation, we summarize the list of known works. Their basic interpretations here are divided into clinical and healthy applications, such that they are accessible to interested readers. Details from each study including study population, task, stimulation parameters, measurement area, primary findings and other information are summarized in Table 3 and visualized in Figure 5.

**Table 3:** Effects of rTMS on fNIRS measures activity during task: clinical and Non-clinical applications.

	**References**	**Population**	**Task**	**Stimulation Parameters**	**Stimulation area**	**Measurement area**	**Sham stimulation**	**No. of Subjects**	**Finding**
Effect of rTMS on Task	Chiang et al., 2007	Healthy	Finger tapping	1 Hz, 115%RMT, 20 min	R-M1	L-M1	Distant secondary coil	5 HC	Task-Evoked [HbO] increase after contralateral TMS for up to 40 min
	Yamanaka et al., 2010	Healthy	Spatial match-to-sample	5 Hz, 100%RMT, 6 s × 10 Trials during Retention period	L/R-PC	Bilateral-DLPFC (Online)	Distant secondary coil	27 Left, 25 Right (HC)	Frontal [HbO] increase after stimulation to P4 during WM task and decreased RT, decrease in [HbO] during control
	Tupak et al., 2013	Healthy	Emotional Stroop	cTBS, 80%RMT, 10 min	L/R-DLPFC	Bilateral DLPFC	Placebo coil	16 Left, 16 Right, 19 Sham (HC)	Bilateral [HbO] decrease to left-PFC stimulation
	Guhn et al., 2014	Healthy	Conditioning stimuli	10 Hz, 110%RMT, 4 s × 40 trains	medial-PFC	Bilateral DLPFC	Placebo coil	40 Active, 45 Sham (HC)	Increased right-medial [HbO] during conditioning, early extinction for exploratory subgroup of strong responders (*N* = 12)
	Yamanaka et al., 2014	Elderly	Spatial Match-To-Sample	5 Hz, 100%RMT, 6 s × 10 Trials during Retention period	L/R-PC	Bilateral-DLPFC (Online)	Distant secondary coil	18 Left, 20 Right (Eld.)	Frontal [HbO] decrease during WM task and increase during Control Task (both P3, P4), no change in RT or accuracy
	Maier et al., 2018	Healthy	Ultimatum and Dictator Game	cTBS, 80%RMT, 40 s	R-DLPFC	Bilateral DLPFC	Placebo coil	19 HC	Reduced [HbO] and generosity during Dictator Game following verum stimulation
Clinical Applications (Task)	Eschweiler et al., 2000	Depression	Mirror Drawing, Mental Arithmetic	10Hz, 90%RMT, 10s × 20 trains, 5 Days	L-DLPFC	Bilateral DLPFC	Tilted coil	12 MDD	Pre-intervention [HbT] change at F3 during left-handed Mirror Drawing correlated with HAMD change
	Dresler et al., 2009	Panic disorder	Emotional Stroop	10Hz, 110%RMT, 4 s × 40 trains, 15 Days	L-DLPFC	Bilateral DLPFC	N/A	Case Study (PD)	Increased bilateral [HbO] to panic stimuli
	Schecklmann et al., 2014	Tinnitus	Audio Stimulation	cTBS, 30% Stimulator Output, 2 sessions × 600 pulses × 5 days	L-TPC	L/R-TPC	Flipped coil	23 Tinnitus (11 Sham), 12 HC	Trend significance toward increased [HbO] in Left-TPC after stimulation
	Deppermann et al., 2014	Panic disorder	Verbal Fluency	iTBS, 80%RMT, 2 s × 200 trains, 15 Days	L-DLPFC	Bilateral DLPFC	Flipped coil	44 PD (23 Sham), 23 HC	No change for real stimulation, [HbO] increase in left-IFG in PD Group following 15 session sham stimulation
	Deppermann et al., 2016	Phobia	Emotional Stroop	iTBS, 80%RMT, 2 s × 200 trains	L-DLPFC	Bilateral DLPFC	Tilted coil	41 Phobia (19Sham), 42 HC (19Sham)	Decreased [HbO] in left IFG for neutral words in phobia group independent of iTBS
	Sutoh et al., 2016	Bulimia nervosa	Food Presentation, Rock Paper Scissors	10Hz, 110%RMT, 5 s, × 15 trains, 5 Days	L-DLPFC	Bilateral DLPFC	N/A	8 BN	Decrease in Left-PFC [HbO] during RPS and neutral stimuli
	Deppermann et al., 2017	Panic disorder	Emotional Stroop	iTBS, 80%RMT, 2 s × 200 trains, 15 Days	L-DLPFC	Bilateral DLPFC	Flipped coil	44 PD (23 Sham), 23 HC	Increased [CBSI] following active stimulation for panic-stimuli
	Hara et al., 2017	Stroke-aphasia	Word repetition	{1 Hz, 40 min}, {10 Hz, 12 min}, 90%RMT, 10 Days	R-IFG	Bilateral-IFG	N/A	4 Left, 4 Right (Stroke)	LF stimulation reduced contralateral [HbO], HF stimulation increased bilateral [HbO]
	Urushidani et al., 2018	Stroke-paresis	Finger flexion/extension	1 Hz, 90%RMT, 20 min, 21 Sessions, 15 Days	Unaffected-M1	Bilateral-M1	N/A	Case study (Stroke)	Increased Lateralization Index (LI) toward lesional hemisphere
	Tamashiro et al., 2018	Stroke-paresis	Finger flexion/extension	1 Hz, 90%RMT, 20 min, 21 Sessions, 15 Days	Unaffected-M1	Bilateral-M1	N/A	59 Stroke patients	Pre-TMS Lateralization predicted changes in treatment lateralization and functional outcomes

### Effects in Healthy Populations

In one of the first studies to examine the effects of TMS on task-evoked fNIRS activity, Chiang et al. (2007) measured the effect of 20 min of 1 Hz stimulation at 115%RMT on evoked hemodynamic changes during a finger tapping task (involving the contralateral motor cortex) for up to 2 h after stimulation. The authors observed an increase in tapping-evoked [HbO] which lasted for up to 40 min after stimulation. While the employed paradigm is expected to induce inhibition in the targeted area, the authors noted that there is no consensus regarding changes to the contralateral motor region with both inhibitory and excitatory effects having been previously reported. The authors theorized that inhibition in the stimulated motor cortex may have increased excitation in the contralateral cortex. Despite the apparent simplicity of the task involved, these findings suggest a particular complexity in the effect of rTMS on task activity, with ostensibly inhibitory stimulation resulting in an increased level of activity in the contralateral cortex. This uncertainty regarding the local and regional effects of rTMS stimulation on fNIRS measures underlies some of the challenges which such studies face, particularly as the task and experimental questions involved increase in complexity.

TMS is commonly applied in neurophysiological studies as a method of interrupting active cognitive processing to understand the function of a particular region. The use of TMS in this context relies heavily on the particular area targeted and its theoretical role in the process investigated, along with when and how the region is stimulated. Here, the goal is primarily to provide causal/diagnostic information to inform further experimental and theoretical models. Regions stimulated with TMS may decrease or inhibit behavioral performance when the region is critical to task execution, or may increase task performance, either by potentiating a processing area, inhibiting an interfering process, or by some other possible pathway. Presently the use of fNIRS during intra-task TMS stimulation has only been investigated by one group under the context of spatial working memory. Yamanaka et al. (2010) studied whether short rTMS bursts of 5 Hz stimulation to the left parietal cortex (PC) during the retention period of a spatial working memory task (delayed match-to-sample) would increase performance. fNIRS was measured simultaneously on the bilateral-DLPFC while stimulation occurred in the adjacent left or right PC. While it was not observed that rTMS stimulation affected task accuracy, active rTMS applied to the right-PC appeared to decrease the reaction time during the working memory task. Active rTMS to the left-PC was also noted to result in [HbO] changes in the bilateral temporal regions. On the other hand, stimulation to the right-PC initially increased [HbO] in the left-precentral and marginal gyri and then shifted to the right gyri and finally the superior frontal gyrus during the response period. The authors described these changes as an asymmetric behavior of functional connectivity between the stimulated regions and suggested that this asymmetry was partially responsible for the effect observed in the right-PC which was not apparent in the left.

In a follow-up of this work, Yamanaka et al. (2014) explored the use of rTMS for cognitive enhancement in a healthy elderly population. However, after repeating the procedure in this population, right-PC rTMS did not significantly affect task reaction time or accuracy. When examining the fNIRS data, the authors observed that, in comparison with the young participants, older participants were observed to recruit more resources as evidenced by higher [HbO] during the working memory task. However, active rTMS had the effect of decreasing the task-evoked prefrontal [HbO] changes accompanying task performance. The authors suggest this is in line with reports that elderly individuals may require higher levels of neural resources in order to achieve similar performance outcomes and that active rTMS may have increased the neural efficiency of these systems. If the elderly participants were indeed engaging maximal cognitive resources, the ability of active TMS to further increase performance by increasing [HbO] may be hampered as the circuits may effectively be “at capacity.” Importantly, the authors again noted a difference in the effect of left and right PC stimulation. While TMS to the right-PC appeared to result in little prefrontal change in elderly participants, TMS to the left-PC activated a similar number of channels, but with spatial differences. These results indicate not only the importance of TMS-fNIRS techniques in elucidating the differences between neural function among populations of interest, but also exemplify the more dynamic nature of stimulation responses during task activity.

As an exploration of rTMS to provide a clinical treatment for anxiety, Tupak et al. examined the Emotional Stroop task in healthy subjects after inhibitory cTBS application to the left or right DLPFC (Tupak et al., 2013). Here, the authors demonstrated that cTBS to the left-DLFPC (but not right-DLPFC or sham) bilaterally reduced task-evoked [HbO] to both neutral and anxiety words. This work appears to offer confirmation that inhibitory effects of cTBS as observed in the motor cortex may also inhibit task-evoked neural activity when applied to the left-DLPFC.

In another study examining a potential therapeutic avenue for anxiety and phobia, Guhn et al. (2014) applied high frequency stimulation to the medial PFC following a fear conditioning session (audio of a scream) in healthy subjects. Active but not sham stimulation, reduced arousal in terms of fear-potentiated startle and skin conductance response during the extinction-learning phase, however, fNIRS measures were not significantly different between experimental groups. When authors selected an exploratory subset of participants who most strongly possessed a conditioned fear response, they identified that the active TMS group evoked a higher [HbO] response to early aversive stimuli in the medial prefrontal channels during the extinction-learning phase. They described these changes as an enhancement of self-regulatory inhibition in response to fear, hoping that such therapy may assist others in their ability to address the challenges of phobia.

Most recently, Maier et al. (2018) used inhibitory cTBS at 80%RMT applied to the right-DLPFC in order to reduce cognitive control over forgiveness. Based on previous studies which had implicated right-DLPFC activation in pro-social reactions to unfair situations, the authors replicated a protocol involving an ultimatum game against unfair or fair opponents, followed by a dictator game in which subjects were able to retaliate (Brüne et al., 2013). Following verum inhibitory rTMS, participants allocated less money to unfair opponents and showed reduced [HbO] in the right-DLPFC compared with sham stimulation. This work may serve as a useful example of how TMS-fNIRS may be used within cognitive neuroscience to investigate neural underpinnings of social interaction and behaviors.

### Clinical Applications

A primary motivation to study the fNIRS-measured effects of rTMS during specific tasks is that fNIRS may offer a useful metric for treatment response, clinical status, or functional targeting for TMS therapies. In one of the earliest works employing both techniques, Eschweiler et al. (2000) reported that functional activation during mental tasks (mental arithmetic and left/right-handed mirror drawing) could serve as a predictor of TMS therapeutic effect in Major Depressive Disorder (MDD). Clinical improvement to 5-days of rTMS therapy (10 Hz, Left-DLPFC) was correlated with pretreatment [HbT] changes in the left-DLPFC during a left-handed mirror drawing task. Given the excitatory nature of high frequency rTMS and expected reduced left-PFC activity in depression, these results appear to confirm a general hypothesis that excitatory rTMS therapy could target regions of relative hypoactivity. As only pre-TMS task measurements were conducted, it was unknown what changes successful therapy may have had on fNIRS activity. However, this work establishes a precedent for functional targeting of TMS therapy using fNIRS-measured activity during tasks which have been identified as affected in a given disorder. Together with the recently published work by Shinba et al. (2018), these studies make a compelling case that the ability or inability of excitatory rTMS to evoke hemodynamic activity may serve to inform clinical status and predict treatment response to TMS therapies.

Dresler et al. reported a case study in which a patient with comorbid panic disorder (PD) and MDD was treated with high-frequency rTMS of the left-DLPFC over the course of 3 weeks (Dresler et al., 2009). fNIRS measures of an emotional Stroop task featuring panic-related and neutral words showed that therapy increased bilateral prefrontal recruitment in terms of [HbO] during exposure to the panic task-condition. These results inspired a larger clinical investigation into the replicability of hypofrontality in PD and the use of excitatory iTBS over the left-DLPFC as a potential treatment approach. In a double-blinded sham-controlled study with 44 patients, the authors separately confirmed fNIRS-measured prefrontal hypoactivity in PD during both a verbal fluency task (Deppermann et al., 2014) and the emotional Stroop task (Deppermann et al., 2017). Following 15 sessions of iTBS treatment and Cognitive Behavioral Therapy (CBT), results indicated that active rTMS did not impact pre-therapeutic activity on the fluency task, but in a confirmation of the original case study, increased the relative activation of panic-related stimuli to neutral stimuli during the emotional Stroop task. Although the work shows a promising indicator that TMS treatments can remediate clinically relevant functional deficits as measured by fNIRS, the link between this functional restoration and clinical effect is less clear due to lack of a significant difference in clinical improvement between sham and active iTBS therapies.

Deppermann et al. (2016) employed iTBS again as a potential remediation strategy in individuals with spider phobia after identifying a hypofrontality in the left-DLPFC during neutral words during an emotional Stroop task in participants with phobia. However, following both active and sham iTBS targeting the left-DLPFC and a virtual reality phobia challenge, functional activation differences between control and phobia participants disappeared. The authors speculated that the mild phobia challenge may have helped recruit a compensatory prefrontal network which normalized activity in phobic participants.

TMS therapies also have the potential to address chronic neurophysiological conditions, such as tinnitus, which otherwise have no clear or effective treatment approach. Tinnitus is a perceptual auditory disorder which has been suggested to correlate with a hyperactivity in the left auditory cortex or abnormal lateralization in response to auditory stimuli. Following this proposed mechanism, TMS has been used as an approach to ameliorate symptoms by using rTMS paradigms thought to inhibit cortical excitability; however, individual variability to treatment remains high. In a sham-controlled exploratory work, Schecklmann et al. (2014) applied cTBS over the course of 5 days to the left Herschel's gyrus and monitored the fNIRS response to audio stimuli before and after the course of treatment. Subjects were exposed to speech noise in a block design paradigm as well as in an event-related paradigm. Overall, results from the study were somewhat contradictory, with active rTMS weakly increasing left-auditory [HbO] in the block design format, but slightly reducing [HbO] in the event-related design task. In addition, sham rTMS therapy exhibited essentially opposite trends in fNIRS activity. Finally, while tinnitus symptoms were successfully reduced after therapy, this reduction was independent of the sham/active condition (Schecklmann et al., 2016).

Clinicians view TMS therapies as a new avenue to improve or complement the efficacy of pharmaceutical and psychotherapeutic approaches, especially in applications where relapse rates are relatively high. Eating disorders represent one such area in which NIBS offer a new hope and fNIRS offers one method to understand the mechanisms behind TMS therapies designed to reduce cravings. In a small population of participants diagnosed with Bulimia Nervosa, Sutoh et al. (2016) measured the left and right DLPFC using fNIRS during a food photo task and a rock paper scissors task in which the participant was asked to intentionally win or lose. Subjects were measured 1 week before, and 4 h after, a single session of 10 Hz rTMS to the left-DLPFC. rTMS was successful at reducing subjective craving of high-calorie food stimuli with no effect on low-calorie or neutral stimuli. This effect was coupled by a reduction of [HbO] in the left-DLPFC to neutral photo stimuli during the latter half of the task. rTMS application prior to the lose/win rock papers scissors task appeared to reduce accuracy during the lose condition which was coupled with reduced [HbO] in the left-DLPFC channel. Although neural responses to desirable foods were not significantly different, the authors suggested that these results reflected an inefficient self-regulatory activity in the PFC which was improved by rTMS, resulting in decreased task [HbO] to neutral stimuli where such self-regulation was unnecessary. Such studies represent important efforts to understand how fNIRS provides insight into the treatment of mental disorders with complex etiologies.

Research into the use of fNIRS to guide and evaluate TMS treatment is still very much in an exploratory phase. However, the accumulation of evidence reviewed here and the increasingly practical application of the two techniques in combination has brought some aspects of this goal closer to reality, in particular, the use of fNIRS as a methodology for functional targeting. Recently, an exciting study used this approach to guide TMS intervention within a population of stroke patients who suffered from chronic aphasia. Hara et al. (2017) divided an exploratory aphasia population with exclusively left-hemisphere lesions into two groups based on the relative hemispheric activity during a word repetition task. Participants who exhibited higher left hemisphere activation were prescribed 1 Hz rTMS to the right inferior frontal gyrus (IFG), whereas subjects with right hemisphere activation were prescribed 10 Hz rTMS to the same location (rIFG). Following 11 sessions of TMS therapy and intensive speech therapy, participants showed significant improvement in language functions as well as differential effects of rTMS therapy depending on the assigned paradigm. Individuals receiving low-frequency stimulation showed a reduction in activation asymmetry, whereas individuals receiving high-frequency stimulation showed an increase in activation within the right hemisphere. Although it is not specifically possible to differentiate the effect of intensive therapy and that of rTMS, this study shows an exciting potential wherein fNIRS may be used to indicate effective TMS approaches and serve as a measure of effective treatment.

Two publications recently have continued this trend by examining the ability of fNIRS to influence or normalize the hemispheric balance after stroke. Described in one case study (Urushidani et al., 2018) as well as a clinical study with 59 participants (Tamashiro et al., 2018), fNIRS was used to evaluate effects of rTMS and occupational therapy on the balance of hemispheric involvement between the affected and unaffected motor cortices following stroke. Prior to and following a 15-day rTMS treatment regime consisting of 1 Hz at 90%RMT to the unaffected motor cortex, motor symmetry was assessed during a finger flexion/extension task. The case study described that 1 Hz rTMS was successful in increasing lateralization index (LI) in the direction of the lesioned hemisphere and the clinical study supported the beneficial nature of this by reporting that changes in LI were correlated with clinical improvement. However, it was also found that affected hemispheric dominance prior to treatment may affect the success of treatment. Despite individual variability in patient response to treatment, fNIRS here offers a clear approach for assessment of post-stroke cortical function as well as a measure of rTMS remediation of potentially deleterious functional asymmetry.

Altogether in this section 16 unique works addressed the ability of rTMS to effect changes on fNIRS activity. Of these, 6 works studied rTMS in tasks with healthy or aged populations. Excitatory rTMS paradigms were reported to increase ipsilateral [HbO] in 2/3 studies and inhibitory stimulation was reported to reduce evoked ipsilateral [HbO] in 2/2 studies. Additionally, 10 clinical works, including 2 case studies, reported the use of rTMS in clinical conditions of depression, phobia, tinnitus, stroke, bulimia, and panic disorder. Currently, significant mismatch in clinical population, task design, stimulation conditions, and other methodological differences greatly restrict the interpretation of these observations. Studies evaluating the effects of rTMS on tasks using fNIRS all reported their findings as changes in [HbO] or as a corrected variant of [HbO] (4/16 studies, CBSI: see Cui et al., 2010), or in terms of hemispheric balance of [HbO] (Lateral Index, 2/16). However, only two studies reported any results in terms of [HbR] or [HbT]. While oftentimes it can be helpful in addressing complicated effects of stimulation on task by focusing on the changes in one biomarker, the absence of other reported measures may make it difficult to replicate and evaluate future findings.

## Relationship Between Cortical Excitability and fNIRS Measures

Although much remains unknown about the relationship between the changes in cortical excitation observed with TMS and measured cortical responses by fNIRS, researchers have explored the use of TMS and fNIRS as complementary probes of neural function. While not considered a neuroimaging technique *per-se*, TMS can provide spatial mapping of cortical regions when tied to MEP amplitude. Mapping the motor region using TMS has been widely used as a way to test the functionality of the motor system in healthy situations and during recovery from traumatic injury such as stroke. Evoked MEP amplitudes are also known to change during task performance and have been proposed as an additional functional measure in some cases. Although these measures require active stimulation and cannot be easily obtained outside of the motor cortex, MEP changes offer an additional perspective on neurophysiological state during cognition. Finally, the combination of fNIRS measures and TMS have been popularized for the study of central fatigue, in particular the effect of hypoxic cerebral and peripheral conditions on exercise and muscle excitability. Although it is readily apparent that hypoxic conditions reduce athletic performance, especially in individuals unacclimated to such conditions, differentiating the causes of degraded performance has not been a straightforward task. Measurement of cerebral and peripheral oxygenation using fNIRS-based systems allows exercise physiologists to independently manipulate and verify the contributions of hypoxic conditions to fatigue under voluntary activity and TMS-evoked muscle movement. Studies describing the relation of fNIRS and cortical excitability are detailed in Table 4 and broken down by the categorized methodological approaches.

**Table 4:** Relation of fNIRS and cortical excitability as measured by TMS: motor mapping, functional MEPs, and central fatigue.

	**References**	**Population**	**Task**	**Stimulation Parameters**	**Stimulation Area**	**Measurement Area**	**No. of Subjects**	**Finding**
Motor Mapping	Park et al., 2004	Stroke-Paresis	Key Turning	110%RMT for Motor Mapping	L/R-M1	Bilateral-M1	Case Study (Stroke)	Decreased [HbO], increased lateralization during task over therapy. Enlarged motor region after therapy.
	Akiyama et al., 2006	Healthy	Hand Grasping	120%RMT for Motor Mapping	L-M1	L-M1	10 HC	Biphasic [HbO] changes observed over MEP COG
	Koenraadt et al., 2011	Healthy	Thumb abduction	120%RMT for Motor Mapping	L-M1	L-M1	11 HC	No difference in [HbO] changes at MEP COG vs C3
MEP during Task	Lo et al., 2009	Healthy	Reading aloud, Singing	110%RMT, random MEP evaluation during task	L-M1	L-M1	5 HC	Changes in evoked MEP amplitude during vocalization at MEP COG, MEP changes not correlated with [HbO]
	Derosière et al., 2015	Healthy	Sustained Attention	MEP evaluated at 5min intervals	L-M1	PFC, L-M1, R-Parietal	15 TMS, 13 fNIRS, 4 Control	Increase in lateral PFC and right parietal [HbO], MEP amplitude increase over TOT
	Corp et al., 2018	Elderly	Dual Task [Tapping, N-Back]	MEP evaluated at 12.5s intervals	L-M1	Exp1: PFC (Online), Exp2: PFC, PM, M1	Exp1: 15 Young, 15 Eld. Exp2: 15 Eld.	Increased CSP during dual-tasks in elderly correlated with worse performance. fNIRS uncorrelated with dual-task performance
Exercise & Fatigue	Millet et al., 2012	Healthy	Isometric elbow contraction	MEP and CSP evaluation every 4th contraction	R-M1	L-DLPFC	Exp1: 12 HC, Exp2: 10 HC	Reduced performance and prefrontal oxygenation in hypoxia despite normoxia in muscle. Similar CSP and MEP responses
	Goodall et al., 2012	Healthy	Cycling	MEP, CSP, VA evaluation at 130%RMT before and after exercise	L-M1	L-DLPFC	9 HC	Reduced performance and prefrontal oxygenation in hypoxia and larger decrease in VA TMS after exercise
	Goodall et al., 2014	Healthy	Cycling	MEP, CSP, VA evaluation at 130%RMT before and after exercise	L-M1	L-DLPFC	7 HC	Greater decrease in prefrontal oxygenation in acute hypoxia, decrease in voluntary and potentiated force in both chronic and acute hypoxia. Doubled MEP size in chronic hypoxia.
	Rupp et al., 2015	Healthy	Isometric knee contraction	MEP, VA, CSP evaluated before and during contractions	L-M1	L-DLPFC	15 HC	Reduced performance in hypoxia, increased prefrontal oxygenation in hypoxia with CO2 clamping vs. w/o, VA TMS decrease greater in hypoxia w/o CO2 clamping
	Jubeau et al., 2017	Healthy	Cycling	MEP, VA, CSP evaluated before and 1,2,3 hours after exercise	L-M1	L-DLPFC, L-M1	10 HC	No performance difference, VA TMS or CSP after prolonged exercise in hypoxia conditions, reduced prefrontal oxygenation in hypoxia
	Marillier et al., 2017	Healthy	Isometric elbow contraction	MEP, VA, CSP evaluated before and during contractions	L-M1	L-DLPFC	11 HC	No performance differences between acute, chronic, or normoxia conditions or in VA TMS decline. Longer CSP in hypoxia conditions. No difference in prefrontal or muscle oxygenation
	Laurent et al., 2018	Healthy	Isometric knee extension	MEP, VA evaluated at 140% 'optimal' power, before and during extension, and after task failure	L-M1	L-DLPFC	14 Trained Athletes	No effect of salbutamol intake on VA TMS, prefrontal oxygenation, or task performance
	Solianik et al., 2018	Healthy	2 Hr Speed-Accuracy Motor task	MEP evaluation at 130%RMT before and after task	L-M1	DLPFC	10 HC	Increased MEP amplitude after prolonged activity, decrease in evoked left-prefrontal [Hb] in Stroop task vs. pre-task Stroop, no change for control activity

### TMS Motor Mapping and fNIRS Motor Mapping

In an early case study by Park et al. (2004), changes in the TMS motor map of a patient undergoing constraint-induced therapy for stroke alongside functional changes in a motor task activity as recorded by fNIRS and fMRI. Therapy was associated with bilaterally decreased task-evoked [HbO] and an increased laterality toward the hemisphere associated with the affected hand. These changes were also reflected in an increased TMS motor map area in the same hemisphere, suggesting that improvements in therapy were associated with improved cortical organization in both measures.

The specificity of TMS mapping has also been speculated as a useful way to inform neuroimaging. In particular, TMS has been proposed as one technique to identify areas that contain the peak fNIRS response which may be sensitive to individual variability. fNIRS sensor arrays can be positioned and arranged in many form factors, but most commonly, optode arrangements are placed based on rough anatomical locations such as 10–20 positions. Mismatches between individual anatomy, as well as functional differences within regions, may serve to reduce the sensitivity of specific measured channels to experimentally-relevant cortical changes by simple virtue of non-optimal placement. In one bid to enhance functional sensitivity, Akiyama et al. (2006) attempted to relate the evoked MEP Center of Gravity (CoG) for the Abductor pollicis brevis (APB) muscle with the functional response to a hand-grasping task using an optode arrangement centered on the CoG. The authors reported that the fNIRS oxygenation response was significantly lower in spatial specificity than the functional area estimated by TMS motor mapping but reported a specific spatial specificity for early-phase changes in [HbR] at the CoG. In a later study, Koenraadt et al. (2011) conducted a similar protocol, again mapping the CoG of the right APB and measuring fNIRS activity at the CoG as well as C3 on the 10-20 System. Although they confirmed that C3 was a poor estimation of CoG position with an average error of 19.2 mm, they were unable to identify any differences in the evoked hemodynamic changes during a thumb abduction/adduction task. The authors suggested that TMS-evoked MEP amplitudes and motor-evoked fNIRS may derive from a different physiological basis.

### Changes in Cortical Excitability During Task: Functional MEP Measures

In a line of work attempting to compare MEP measures to task-evoked fNIRS, Lo et al. (2009) investigated the changes in TMS excitability and functional changes while subjects were engaged with overt reading or singing tasks. After identifying the CoG of the FDI muscle, experimenters identified 9 surrounding points at which, during separate sessions, either fNIRS measures or MEP amplitudes were evaluated during the speaking/singing tasks. Changes in the MEP amplitude were observed most strongly at the CoG, whereas fNIRS activation was observed as more distributed increases in [HbO]. The authors reported that maximum fNIRS activity did not occur at the CoG and fNIRS measures did not significantly correlate with the changes in MEP amplitude. Again, the authors claimed that the MEP and evoked [HbO] evaluated distinct neural dynamics involved in vocalization and that suggested that cortical excitability itself does not likely imply high metabolic demands.

Another work by Derosière et al. (2015) examined functional and motor excitability during sustained attention. Subjects were divided into two experimental groups, one group receiving regular MEP measurement and one monitored using fNIRS. The authors worked to show how functional measures in different cortical areas and excitability in the motor region varied with the Time on Task (TOT). During a 30-min performance of a sustained attention task, increases in [HbO] in the lateral prefrontal regions and the right-parietal areas evolved after approximately 9 min of performance, whereas the left-M1 region exhibited these changes after 15 min. In the TMS group, increased MEP amplitude was also evidenced after 15 min of task performance, an effect not present in the non-task TMS control group. Together, the authors suggested that attentional areas including the lateral prefrontal regions and right parietal region may be more sensitive to prolonged attentional demands and that motor regions may become more involved in later stages of the task. Evidence of an increase in MEP amplitude following an extended attention-demanding task was also supported by a more recent study focusing on cognitive changes due to prolonged motor actives. Solianik et al. (2018) investigated changes to fNIRS-evoked cognitive activities and motor function following a 2 h speed-accuracy motor task compared with a non-demanding control task. Following the motor task, the authors noted increased prefrontal oxygenation (driven by decreased [HbR]) along with an increased resting MEP, whereas the non-demanding control task exhibited no changes on either cognitive biomarkers or cortical excitability. Most recently, MEP amplitudes and the cortical silent period (CSP) length were evaluated as a measure of engagement during dual-task performance in elderly and adult populations (Corp et al., 2018). While increased CSP length was associated with poor performance in elderly subjects, fNIRS measures were excessively noisy during an initial experiment and did not correlate with behavioral performance in a second experiment.

Although the preceding studies did not examine the effect of TMS on fNIRS measures but rather the relation between TMS measures and fNIRS, they still help inform works which do examine these approaches. In particular, these works emphasize the spatial dissimilarity between MEP measures and task-evoked fNIRS. In the context of motor mapping, observation of a broader fNIRS response may be interpreted as additional recruitment involved in voluntary muscle control as compared with TMS-evoked movement at rest. In the context of active vocalization, the dissimilarity in FDI excitability with the functional activity of the task itself may reflect different roles between motor control, planning, and cortical excitation during task execution. On the other hand, regional similarities in the emergence of time-on-task effects during prolonged attention suggest some common roles for the two cortical measures. Importantly, these works together suggest that TMS may activate more spatially specific areas than those recruited in voluntary activities which may be the result of more coordinated neural activities, even for relatively simple tasks.

### Changes in Cortical Excitability During Exercise, Hypoxia, and Central Fatigue

In first work of this series, Millet et al. (2012) monitored muscle and cerebral hemodynamics using fNIRS under differing fixed inspired oxygen (FiO2) levels while the muscle performing contractions was occluded using an inflated cuff so that systemic hypoxia could be maintained during local normoxia. After occluding the arm, inspired oxygen was then administered for 5 min and afterwards subjects performed repeated isometric contractions until exhaustion. Electrical nerve stimulation (M wave) and TMS were used to generate muscle twitches during isometric contraction performance and assess the relation between spinal and cortical inhibition. Under severe hypoxia, muscle performance was decreased by 15% despite similar muscle hemodynamic conditions, but electrically and TMS-evoked MEP and CSP were unaltered by hypoxia, suggesting that reduced oxygenation in the brain may have a role in this performance reduction.

Extending this work by exploring the additional contribution of hypocapnia on central fatigue, Rupp et al. (2015) used the combination of TMS and fNIRS during knee extensions with CO_2_ clamping to control end-tidal CO_2_ concentrations. Reductions in exercise performance in terms of duration were again noted in hypoxic conditions regardless of the presence of CO_2_ clamping conditions. However, CO_2_ clamping appeared to increase both cerebral and muscular oxygenation. Additionally, maximal voluntary activation by TMS (VA_tms_) at task failure during hypoxia was larger with CO_2_ clamping. However, electrical muscle stimulation revealed that CO_2_ clamping resulted in increased peripheral fatigue, suggesting that the control of expired CO_2_ to prevent hypocapnia had shifted the balance of fatigue from central to peripheral mechanisms.

The most common environmental hypoxic situations are introduced by high-altitude conditions which can influence certain sports such as hiking and cycling. In two works, Goodall et al. (2012, 2014) examined the effect of hypoxia on cycling activity first by emulating high-altitude (3,800 m) conditions with changes in FiO_2_ (Goodall et al., 2012), and then in individuals acclimatized to high-altitude conditions (14 days at 5,260 m) (Goodall et al., 2014). In the first study, hypoxia significantly reduced cycling duration as well as cerebral [HbO]. VA_TMS_ activation was further reduced after cycling compared with normoxia, but MEP amplitude was increased after hypoxia. The second work showed that while maximal contraction force, VA_TMS_, and prefrontal oxygenation were decreased by the introduction of altitude-induced hypoxia, adaptation to the high-altitude environment appeared to normalize prefrontal hemodynamics and abolish the observed decrease in VA_TMS_. Additionally, the authors observed that after altitude acclimatization, TMS-evoked MEP amplitudes were nearly twice as large, indicating that the central fatigue was attenuated by acclimatization, possibly through increased cerebral oxygenation or cortical excitability.

In contrast to these findings, Jubeau et al. (2017) studied the effects of hypoxia in cycling during prolonged (80 min) rather than strenuous conditions. During the performance of more moderate exercise, performance differences were not significant between the two conditions, including changes in TMS measures and electrical nerve stimulation. Although prefrontal and motor cortex oxygenation were reduced in hypoxic conditions, the authors suggested that low-intensity prolonged exercise does not result in increased central fatigue. Similarly in another altitude acclimatization study (5 days at 4,350 m), Marillier et al. (2017) noted that exposure to hypoxia did not alter excercise duration, prefrontal hemodynamics, muscle hemodynamics, or voluntary contraction force after low intensity isometric elbow flexion. While electrical nerve stimulation showed increased peripheral fatigue, TMS showed decreased cortical excitability after 5 days at high-altitude, suggesting that despite increased central inhibition, performance of less intense isometric exercise may not be substantially affected by hypoxia-induced central fatigue.

In another inquiry into central fatigue, Laurent et al. (2018) explored the use of TMS and fNIRS to determine the effects of Salbutamol on central fatigue and prefrontal oxygenation in trained cyclists. Knee extensions were performed until task failure at a variety of intensities, however differences were not observed in prefrontal oxygenation, VA_TMS_, or athletic performance after intake of oral or inhaled Salbutamol. This work, while reporting negative results, shows an interesting application of fNIRS-TMS in evaluation of athletic doping. Salbutamol, an asthma medication, has uncertain effects on exercise performance, unlike the often more dramatic changes created by hypoxic conditions, but this study opens the door toward further usage of this methodology in the study of pharmaceutical effects on central fatigue and cortical excitability.

## Limitations

The work presented here attempts to address an absence of a collective discussion of the mechanisms and applications of fNIRS and TMS. The range of applications and uses of the techniques as well as differences between protocols, subject populations, stimuli locations, and stimuli parameters can further exacerbate the lack of standardization between study objectives and approaches. In spite of this, presentation of the unified body of works still allows for an accessible comparison of methodological approaches so that future studies can explore and expand on the results of these findings.

The breadth of topics covered in this review places restrictions on the ability to perform comparative analysis between all studies introduced here. In the first primary section, we grouped together studies in which participants were measured during TMS stimulation under both offline and online contexts, both of which may measure different post-stimulation phenomena attributable to differences in experimental setup. Studies in this section were grouped in a manner according to the location at which stimulation was performed; however, differences between TMS coils used, targeting approaches, statistical/preprocessing approaches, as well as fNIRS equipment and measurement locations could introduce significant variability to the results expressed in the reported studies. While it is difficult to provide a quantitative comparison between all studies presented here due to this, some similarity in works may allow for a preliminary generalization of findings for expected activity to the most common stimulation parameters in M1 and the DLPFC. To this end we have made a qualitative evaluation of ipsilateral and contralateral responses to stimulation available in Supplementary Table 1. Strict review of the studies presented here also shows that a large number of studies originate from only a few groups which may impair the independence of reported findings.

In the second section, fNIRS was primarily used to investigate changes during task performance which could be attributed to rTMS stimulation. Within this designation, fNIRS is at times used as either a method to detect changes in evoked measures during task performance due to rTMS therapy in clinical populations, or as a method for modulating cognition within healthy individuals. Studies such as these may depend largely on the types of the tasks employed, as well as the clinical condition being targeted. These interventions may also differ in terms of their targeted stimulation/measurement regions, parameters and the length or style of intervention. For this reason, quantitative comparison of studies cannot yet be made, but the current progress within the conjoined application of the two modalities is discussed.

The third section concerns the use of fNIRS and cortical excitability within a functional and physiological context. This section contains several novel approaches for the use of MEP as functional measure in itself, but also describes the relationship of cortical oxygenation to the central nervous system's role in neuromuscular fatigue. Work involving the functional MEP measures allows a general comparison of changes in MEP excitability with evoked functional measures. However, it is generally understood that the physiological basis of both measures is very different in nature and as such, experimental designs to compare these measures must be constructed in a manner which takes advantage of the unique aspects of each measurement approach.

The preliminary nature of many of the works included in this review, as well as the limited number of researchers investigating these topics, precludes a rigorous investigation of bias. It is not currently clear whether reports where fNIRS measures, or fails to measure, the effects of TMS may be due to the stimulation parameters or the methodology used. Apart from these mentioned limitations, there are many other variables which govern the influence of rTMS on normal neurophysiology including anatomical differences and subject variability in response to rTMS. The state-space of TMS effects on neurophysiology may have an incredible complexity and considerable work must be done in order to consolidate the effects of even simple paradigms.

## Summary and Future Directions

The combination of TMS and fNIRS as paired techniques for the study of neurophysiology and cognition has expanded well beyond the work of a handful of researchers. Given the mutual advantages of the techniques and the individual proliferation of both technologies in terms of availability to clinicians and procurement by researchers, the convergence of the fNIRS-TMS is easily anticipated. Currently, scientists have only just begun to employ fNIRS-TMS and many areas of research with rather rich application remain uninvestigated or under-investigated. This includes, but is not limited to, the basic responses to individual pulses in different cortical regions, short trains of rTMS stimulation, effects of prolonged therapies, and investigation in clinical populations. In order to encourage further research and formalize the available knowledge, this review attempts to consolidate current findings regarding the effect of TMS on fNIRS measures under conditions of both rest and task. Furthermore, this work aggregates research into the spatial and functional relationship between fNIRS and cortical excitability measures.

### Effects of TMS at Rest: DLPFC & M1

Here we have discussed results from studies of stimulation broadly in multiple cortical regions and the effects observed from various rTMS patterns with the goal of strengthening core findings by aligning similar investigative works. Studies have most frequently examined either the DLPFC or M1 as candidates for online stimulation and measurement; however, there exist a number of inconsistencies in the results which may be attributed to methodological variations, experimental error, subject variability, and other issues. Studies have illustrated that stimulus intensity, subject state during stimulation, location, and frequency all have an influence on the measured hemodynamic response in different regions. Some consensus exists that short trains of 1 Hz stimulation may reduce [HbO] in both the DLPFC and M1 regions. However, differences between these regions may exist for Single Pulse stimulation responses. Several studies seem to support the finding that subthreshold single pulses to M1 can increase [HbO] in a state-dependent measure, and on the other hand, research into responses in the DLPFC seem to indicate that suprathreshold Single Pulse stimulation decreases [HbO] while subthreshold stimulation does not effect a measurable response. This effect has been previously attributed to either differences in physiology in between M1 and the DLPFC in response to stimulation (Bestmann et al., 2008), greater scalp-cortex distances, or increased sensitivity of M1 (Thomson et al., 2011b). While this dichotomy is intriguing, it should be taken with a grain of salt. Notably, due to the primarily exploratory nature of these studies, many typical stimulation conditions have not been evaluated in a balanced manner.

There is a need for improved experimental control and repeatability in these studies with distinct lack of replication by independent research groups. Fortunately, both rTMS and fNIRS have substantially changed and improved over the past decade, with refinements in hardware, signal processing, sensors, and neuronavigated targeting, allowing researchers and clinicians more fine grain control over their stimulation systems. It is especially important that these new tools are used to translate discoveries from the behavior of cortical excitability changes to improvements in actual clinical applications as rTMS response rates, while significant, average 30–40%. Future TMS-fNIRS studies should consider focusing on the relationship between cortical activities in the motor cortex and the DLPFC.

### Effects of rTMS on Task: Clinical and Non-clinical Applications

As cognitive and clinical neuroscientists seek to employ brain stimulation as a research tool and therapeutic approach, a substantial need for objective and quantifiable measures of stimulation effects presents itself. Several studies have used fNIRS to monitor or describe changes in task activity following rTMS. In clinical and non-clinical studies, rTMS has been successfully used to enhance or suppress cortical involvement with the aim of altering behavioral performance and clinical outcomes. Recently promising studies have provided preliminary evidence that rTMS may guide a cortical reorganization of functional activity following disorders such as stroke. Here, fNIRS offers a technique to monitor the efficacy of rTMS therapy, but also potentially identify treatment targets and stimulation parameters. Unfortunately, the limited number of clinical studies currently available prevent clear interpretation on the measured effects of rTMS paradigms as well as the clinical implications of such effects due in part to lack of replicated works, as well as incomplete reporting of affected fNIRS biomarkers. Despite this, future works may build upon these studies to provide explicit treatment recommendations informed by neuroimaging.

### fNIRS and Cortical Excitability

The study of fNIRS functional measures and TMS-evoked MEPs represent a different, but important role for hybrid TMS-fNIRS with particular utility in functional mapping and the role of central fatigue in exercise physiology. Although this topic represents a smaller portion of the research covered here, since the effects of rTMS are often assessed with respect to changes in RMT, the influence of rTMS on fNIRS measures may require a deeper understanding of the relationship between cortical excitability and neurovascular coupling. Primarily these works identify a broader fNIRS response to voluntary motor activities as compared to regional mapping with TMS. These differences may represent functional differences related to motor control, planning, and other component processes during task execution. While it is expected generally that the ability of TMS to excite specific motor pathways might be more localized than activity related to voluntary motor movements, observations reviewed here show some similar functional trends between MEP measures and fNIRS measures during task execution. These may suggest some common roles between the two measures which may merit further investigation.

## Conclusion

TMS-fNIRS as a multimodal strategy for imaging and cortical interrogation compliments the perspectives offered by TMS in combination with fMRI and EEG for the study of cortical changes in excitability, inhibition, and connectivity. This multimodal approach may even be expanded such that TMS-fNIRS may be deployed alongside EEG or fMRI, or with additional stimulation approaches such as TES for more complex, but complete, assessment and treatment. While scientific works add to a growing body of knowledge, in parallel, technological challenges may be remediated through improved sensor design, optode montages, signal processing, and coil design, altogether enhancing the power and utility of the technique. As works here have presented, fNIRS as a methodology is well-equipped to monitor both transient and prolonged effects of TMS, but as of yet, the available research is limited in its replication and scope. This need for further work should not be used to dismiss the opportunity and unique information which may yet be afforded by TMS-fNIRS for scientific investigation, adaptive therapy, as well as prognostic and diagnostic applications.

## Author Contributions

HA, ST, and JS designed the structure and scope of the review. AC aggregated reviewed articles and prepared the draft manuscript. All authors reviewed and revised the manuscript.

### Conflict of Interest Statement

The authors declare that the research was conducted in the absence of any commercial or financial relationships that could be construed as a potential conflict of interest.

## Supplementary Material

Supplementary Table 1 Detailed list of stimulation parameters and qualitative results for all studies investigating stimulation at rest.

Supplementary Table 2 Detailed list of stimulation parameters and qualitative results for all studies investigating the effects of stimulation on task.
